# Unraveling a 150-Year-Old Enigma: *Psalidodon rivularis* (Acestrorhamphidae: Acestrorhampinae), a Species Complex or a Polymorphic Species?

**DOI:** 10.3390/biology14121793

**Published:** 2025-12-16

**Authors:** Igor Henrique Rodrigues-Oliveira, Priscila Martins de Assis, Luiz Guilherme Pereira Pimentel, Rafael Augusto Silva Soares, Iuri Batista da Silva, Renan Rodrigues Rocha, Fabiano Bezerra Menegidio, Rubens Pasa, Karine Frehner Kavalco

**Affiliations:** 1 Institute of Biological Sciences, Federal University of Minas Gerais, Belo Horizonte 31270-901, MG, Brazil; luizpimentelbio@gmail.com (L.G.P.P.); iuri.b.s@hotmail.com (I.B.d.S.); 2 Laboratory of Bioinformatics and Genomics, Federal University of Viçosa, Rio Paranaíba 38810-000, MG, Brazil; priscila.assis@ufv.br (P.M.d.A.); rafaelaugusto220699@gmail.com (R.A.S.S.); rpasa@ufv.br (R.P.); 3 Laboratory of Ecological and Evolutionary Genetics, Federal University of Viçosa, Rio Paranaíba 38810-000, MG, Brazil; 4 Technological Research Center, University of Mogi das Cruzes, Mogi das Cruzes 08780-911, SP, Brazil; rodriguesr2010@gmail.com (R.R.R.); fabianomenegidio@umc.br (F.B.M.)

**Keywords:** cytotypes, cryptic species, phylogenomics, morphometrics

## Abstract

*Psalidodon rivularis*, a fish endemic to the São Francisco River Basin and known as “piaba do córrego,” has long been regarded as a widely distributed species complex, exhibiting remarkable morphological and cytogenetic variation, even in sympatry. This study aims to determine whether *P. rivularis* represents a single polymorphic species or a group of cryptic species. We analyzed meristic, morphometric, and karyotypic data from 419 specimens identified as *P. rivularis*, as well as from the related species *Astyanax turmalinensis* and *Hyphessobrycon santae*. Additionally, we inferred the phylogeny of the group using NGS data from 25 individuals, incorporating both mitochondrial and nuclear genomic sequences. Our integrative results support the recognition of at least five distinct species within the *P. rivularis* complex. The true *P. rivularis* (called morphotype 1) has 46 chromosomes, while the others have 50 and differ in both morphology and distribution. One of these corresponds to *Psalidodon santae* comb. nov.—which includes *A. turmalinensis* as a junior synonym—and three others are newly described species. These findings clarify the diversity of fishes in the São Francisco River Basin and highlight the importance of conserving its unique freshwater ecosystems.

## Simple Summary

This study investigates a group of closely related fish species from southeastern Brazil known as the *Psalidodon rivularis* complex. These fishes are very similar in appearance, which has made it difficult to know exactly how many species exist. By combining information from their body shape, body measurements, chromosome counts, and DNA, we found that what was once thought to be a single species includes at least five distinct ones. The true *P. rivularis* has 46 chromosomes, while the others have 50. One of them corresponds to *Psalidodon santae*, which was previously classified in another genus, and three are new species described here. Our results also suggest that their diversity arose through processes such as changes in chromosome numbers, hybridization between populations, and morphological divergence, potentially within broader scenarios that may involve vicariant or adaptive processes. These fishes live in small rivers and streams of the Upper São Francisco River basin, environments that are rich in unique species but threatened by human activities. Recognizing and protecting these new species is essential for conserving the biodiversity of Brazilian freshwater ecosystems.

## 1. Introduction

The genus *Psalidodon* Eigenmann, 1911 was originally proposed by Eigenmann to accommodate a single species: *Psalidodon gymnodontus* Eigenmann 1911. Following the transfer of *P. gymnodontus* to the genus *Astyanax* [1], *Psalidodon* fell into disuse until it was “resurrected” by Terán et al. [2], who provided an expanded diagnosis and included several additional species. Currently, the genus comprises 56 species, most of which were formerly assigned to *Astyanax* Baird & Girard, 1854 (46 spp.), followed by *Hyphessobrycon* Durbin, 1908 (5 spp.). With the inclusion of several species complexes, such as the “*Astyanax*” *scabripinnis* group [3], the number of recognized *Psalidodon* species is expected to increase in the coming years.

Among the species added to the genus *Psalidodon* by Terán et al. [2] is *Psalidodon rivularis* (Lütken 1875) (originally described as “*Tetragonopterus*” *rivularis*). This small characin is considered endemic to the São Francisco River Basin and has recently been recognized as a species complex, based on morphological, cytogenetic, and genomic data. Notably, it exhibits chromosomal differences, with cytotypes of 2*n* = 46 and 2*n* = 50 chromosomes [4]. Morphological variation in the species has been noted since its original description; Lütken [5] reported information provided by Reinhardt, the collector of the specimens, stating that schools of *P. rivularis* often showed distinct local characteristics in body coloration and shape. To document this morphological variation, Lütken included two illustrations, which we refer to here as *morphotype* 1 (plate V: Figure 13 in the original work) and *morphotype* 2 (plate V: Figure 14 in the original work), henceforth M1 and M2, respectively.

Another source of morphological variation mentioned by Lütken [5] concerns the completeness of the lateral line, which in this species can be complete, incomplete, or interrupted. However, the same author cautioned that this feature should not be considered a valid diagnostic character at the species or subspecies level, as it may vary even between the two sides of a single individual. This observation was not considered by Eigenmann [6], who, upon examining a lot containing four types of “*T.*” *rivularis*—two with a complete lateral line and two with an incomplete one—named a new species for the variant with the incomplete lateral line: *Hemigrammus santae* Eigenmann 1907, later *Hyphessobrycon santae* (Eigenmann 1910). He also noted, without specifying, differences in body shape and coloration. Upon examining the types of *H. santae* described by Eigenmann [6], their similarity to *P. rivularis* M2 becomes evident, suggesting that they fall within the hypodigm of this species, that is, the set of documented specimens of a species that record its morphological variation.

A third species that falls within the hypodigm of *P. rivularis* is *Astyanax turmalinensis* Triques, Vono & Caiafa, 2003. This species was originally described from the Jequitinhonha River Basin, with little direct comparison to *P. rivularis* [7], and was later reported from the Doce River and the das Velhas River, in the Serra do Cipó National Park, where it occurs sympatrically with *P. rivularis* [8]. Triques and Queiroz [8] provided a diagnosis distinguishing *A. turmalinensis* from *P. rivularis* based on four morphological characters; however, it appears that their comparison was focused primarily on *P. rivularis* M1. In contrast, similarly to *H. santae*, *A. turmalinensis* is more closely related to *P. rivularis* M2.

The term species complex is commonly used to refer to sets of closely related species that are nearly identical and whose boundaries are difficult to define. Delimiting species within species complexes using only morphological characters can be challenging, if not unfeasible, due to several factors inherent to such complexes, including morphological monomorphism, sympatric occurrence, and potential interbreeding [9]. In the case of fishes associated with the former *P. scabripinnis* complex, one can also cite the absence of discrete gaps in meristic and morphometric data [10]. Moreover, morphological traits can be highly homoplastic, with numerous characters having evolved multiple times within fishes of the Characoidea clade [11]. Examples include incomplete lateral lines and the loss of the adipose fin—traits often associated with miniaturization events—among other features [12].

In this context, an integrated approach that combines phenotypic and molecular data—especially from multiple loci—should be encouraged [9]. Integrative approaches using multiple data sources, such as DNA barcoding, cytogenetics, morphometrics, and morphology, have been successfully applied in recent studies to delimit species in problematic groups such as *Astyanax lacustris* and species of the *P. scabripinnis* complex from Rio Grande do Sul, Brazil [10,13].

In this study, our aim was to determine whether the morphological and karyotypic variation observed in *Psalidodon rivularis* sensu lato corresponds to a highly polymorphic species or to distinct cryptic and semi-cryptic species occurring sympatrically in the Upper São Francisco River Basin. Here, we define *P. rivularis* sensu lato as all specimens that fall morphologically within the hypodigm of the species, based on its original description [5], which consequently includes individuals previously attributed to *H. santae* and *A. turmalinensis* [14]. To address this question, we employed an integrative approach that combined phenotypic data (karyotype, morphology, and morphometrics) with genomic data (mitochondrial DNA, or mtDNA, and various orthologous loci obtained from Next-Generation Sequencing, or NGS, short-read data).

## 2. Materials and Methods

### 2.1. Sampling

**Figure 1:**
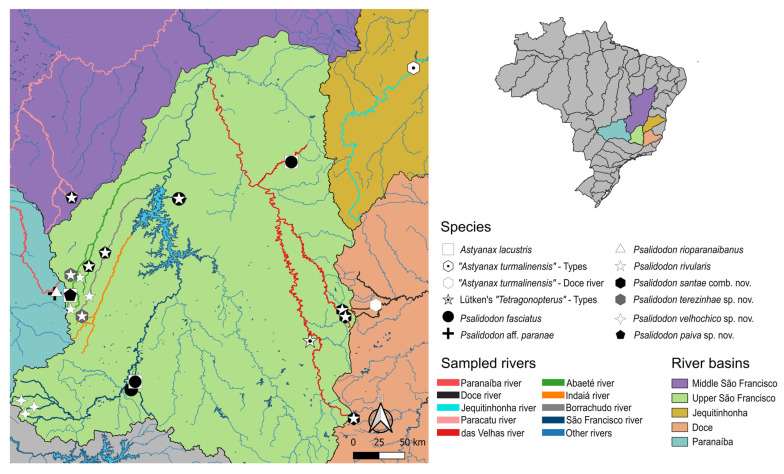
Map of the study region showing the sampling sites used in this study for *Psalidodon rivularis* “sensu lato” and comparative material. Two overlapping sampling sites in Serra do Cipó National Park are not visible. The “*Astyanax turmalinensis*” specimen from the Doce River basin was not included in the present study but is shown on the map to illustrate the occurrence of the *P. rivularis* “sensu lato” group in the Doce River basin.

For this study, we analyzed a total of 473 specimens, of which 419 corresponded to *Psalidodon rivularis* sensu lato. The specimens were collected from 26 different localities, the majority of which (22 sites) are in the Upper São Francisco River hydrographic mesoregion (Figure 1). Of this sampling effort, morphological, molecular, or karyotypic data were obtained from 260 specimens of characins collected specifically for this study between 2021 and 2024. An additional 162 specimens were obtained from individuals previously deposited in the Ichthyological Collection of the Laboratory of Ecological and Evolutionary Genetics (LaGEEvo), Federal University of Viçosa–Rio Paranaíba Campus (UFV–CRP). These individuals, including some outgroups, were collected between 2008 and 2024, and their tissue samples and cell suspensions are stored in the Tissue and Cell Suspension Bank of the same laboratory.

We personally analyzed morphological and morphometric data from 20 specimens of the type series of *Astyanax turmalinensis* (holotype + 19 paratypes) deposited in the ichthyological collection of the Department of Zoology at the Federal University of Minas Gerais (DZUFMG). Our analyses also included high-resolution photographs and Computed Tomography (CT) scans of the lectotype of “*T.*” *rivularis*, as well as syntypes of *Tetragonopterus lacustris* (Lütken, 1875) (valid name: *Astyanax lacustris*) and *Tetragonopterus cuvieri* (Lütken, 1875) (valid name: *Psalidodon fasciatus* Cuvier, 1819), all made available on the website of the Natural History Museum of Denmark’s biological collections (https://collections.snm.ku.dk/en (accessed on 17 September 2024)). Finally, we also examined photographs and notes generously provided by Professor Carlos Alexandre Miranda Oliveira, including specimens such as syntypes of *H. santae* and paralectotypes of *P. rivularis* previously analyzed in his thesis [14]. Detailed information on the specimens analyzed in this study can be found in Supplementary Material S1.

### 2.2. Sampling and Collection of Tissues and Cell Suspensions

For this study, we conducted field sampling at 15 sites across the Upper São Francisco River hydrographic mesoregion, under permits issued by SISBIO (Biodiversity Authorization and Information System, license number 25634-9) granted to Karine Frehner Kavalco. Specimens were collected primarily using passive methods, employing 80 cm diameter funnel traps. As bait, we used small 3 cm^3^ cubes of red meat mixed with flaked wheat flour or ground bread. Alternatively, in some locations, active sampling was employed using cast nets, hand nets, dip nets, or gillnets with 15 mm mesh size.

After collection, the animals were transported in buckets filled with clean water and equipped with aerators to LaGEEvo UFV CRP, where they were euthanized for tissue and cell suspension extraction, and subsequently fixed for deposition in the ichthyological collection. Specimens transported to LaGEEvo UFV CRP were kept in 25 L aquaria equipped with water filters, aerators, and thermostats set to 27 °C, where they remained until the day of euthanasia. The only exception was the specimens collected in the Serra do Cipó National Park, which were euthanized and processed within the park premises.

The specimens were euthanized in 1% Eugenol, following the guidelines of Normative Resolution No. 37 of the National Council for Animal Control and Experimentation, of the Ministry of Science, Technology, Innovations and Communications (CONCEA-MCTI). All experiments with live animals performed in this work were conducted after approval of the project submitted to the Ethics Committee on Animal Use of the Federal University of Viçosa (CEUA-UFV) (protocol: 23/2023).

After specimen collection, heart and liver tissues were extracted and stored in 1.5 mL Eppendorf tubes containing absolute ethanol for subsequent DNA extraction. For the preparation of cell suspensions, spleen, anterior kidney, and posterior kidney tissues were collected, following a protocol adapted from Bertollo [15]. We use the medication Broncho-Vaxom^®^ for immunological induction between 24 and 48 h before euthanasia [16]. The detailed protocol can be found in Supplementary Material S2.

### 2.3. Karyotype Preparation, DNA Extraction, and Genomic Analyses

To visualize metaphase chromosomes, we dropped the cell suspensions onto glass slides and proceeded with staining using 10% Giemsa diluted in phosphate buffer (pH 6.8). Chromosome morphology was characterized based on the ratio between the lengths of the long and short arms, following the classification proposed by Levan et al. [17], using the software IdeoKar v.1.3 [18].

In the genomic analyses, we extracted DNA from 13 specimens of *P. rivularis* sensu lato collected from different sites in the Upper São Francisco River basin (12 specimens) and the Middle São Francisco River basin (1 specimen), as well as from one specimen of *Psalidodon* aff. *paranae* collected in the Paranaíba River basin. The *P. rivularis* sensu lato individuals were sampled from eight distinct localities in the upper and middle São Francisco River basin, covering tributaries of six major rivers (Abaeté, Borrachudo, Indaiá, Paracatu, São Francisco, and das Velhas) and representing all morphotypes identified in this study. Most of the specimens (11) had been previously karyotyped.

To obtain DNA for sequencing, we developed an adapted protocol using the Quick-DNA/RNA Viral Magbead kit (Zymo) and the Loccus Extracta 32 automated extractor. The protocol used is detailed in Supplementary Material S3. After extraction, we assessed DNA integrity on a 1% agarose gel and evaluated its purity using a Thermo Scientific™ NanoDrop Lite Plus spectrophotometer (Thermo Fisher Scientific, Waltham, MA, USA). The samples were sequenced on the Element Biosciences Aviti System platform (Department of Biosystems Science and Engineering, ETH Zurich, Basel, Switzerland), generating 2 × 150 libraries for each sample with up to 5 Gb of data.

For the phylogenetic reconstructions, we considered two sources of data: mitochondrial genome (mtDNA or mitogenome) and orthologous fish genes (training dataset: *Danio rerio*) predicted using the Augustus tool v.3.4.0 [19]. To perform assemblies, annotations, alignments, and phylogenies, we used the Galaxy Europe platform [20]. In addition to the 14 libraries we sequenced, we included 11 additional samples in our analysis. These samples comprise eight previously assembled and annotated mitochondrial genomes (*Astyanax aeneus*, *Astyanax altiparanae*, *Astyanax lacustris*, *Astyanax mexicanus*, *Psalidodon fasciatus*, *Psalidodon rioparanaibanus*, and two samples of *P. rivularis*) from the study by Pasa et al. [21], the mitochondrial genome of *Psalidodon paranae* [22], and two mitochondrial genomes that we assembled from National Center for Biotechnology Information Sequence Read Archive (NCBI SRA) libraries (*Psalidodon scabripinnis*: SRR9985989 and *Psalidodon correntinus*: SRR11147340).

We assembled the mitogenomes using the software NOVOPlasty v.4.3.1 [23], except for one sample (*P. rivularis* from Córrego Crico, a sub-basin of the Paracatu River), for which we used GetOrganelle v.1.7.7.1 [24]. The K-mer values and seed sequences used can be found in Table 1. The mitochondrial genomes were annotated with MITOS2 v.2.1.9 [25], available on the Galaxy Europe platform, using the genetic code 2 (vertebrate mtDNA) and the RefSeq89 (Metazoa) database, and also with MitoAnnotator, from the MitoFish platform [26]. For phylogenetic analyses, we extracted and used the protein-coding genes (PCGs) and ribosomal RNA genes (rRNAs).

To estimate the phylogenetic relationships of the group using a data source different from the mitogenome, we performed phylogenomic analyses with orthologous PCGs recovered in all libraries or in at least 90% of them. In addition to the libraries we submitted for sequencing and the previously mentioned libraries of *P. scabripinnis* and *P. correntinus*, we used the libraries of *P. paranae* (SRR5461471), *A. aeneus* (SRR1927238), *A. mexicanus* (SRR2040423), and the remaining libraries from the study by Pasa et al. [21].

To obtain these orthologous loci from raw short-read data, we followed the protocol of Roncoroni and Gallone [27] with some modifications, using a workflow implemented in the Galaxy Europe platform [20]. Supplementary Material S4 describes the modified protocol, including references for each tool used. The pre-annotation steps of the PCGs in the protocol involve: quality trimming, adapter removal and evaluation of the result with the Trimmomatic v.0.39 and FastQC v.0.74 tools [28,29], assembly of contigs with the Megahit v.1.2.9 tool [30], removal of isoforms with cd-hit v.4.8.1 [31], evaluation of the quality of the assemblies with Fasta Statistics v.2.0 and Busco v.5.5.0 [32,33], renaming of fastas with Replace Text v.9.3 [34] and masking of repetitive regions with RepeatMasker v.4.1.5 [35]. After annotating the PCGs in each library with Augustus tool v.3.4.0 [19], we concatenated all the resulting sequences into a single dataset and filtered this dataset to retain only sequences with a minimum length of 300 nucleotides. Only after this filtering step did we perform the ortholog search using the tool Proteinortho6 v.6.3.4 [36].

For both mitochondrial genes and orthologous PCGs, each gene was aligned using MAFFT v.7.526 [37], with the FFT-NS-2 strategy applied to mitochondrial genes and the L-INS-i strategy used for orthologous genes. After alignment, the genes were concatenated and partitioned using the Concatenator tool v.0.3.1 [38]. For the PCGs, partitioning was performed for both codon position and gene, while for rRNAs, partitioning was based only on the genes. Three concatenated datasets were used: mtDNA (mitochondrial PCGs + rRNAs), 100% matrix (orthologous PCGs recovered in all libraries), and 90% matrix (orthologous PCGs recovered in at least 23 libraries). We used the tool ClipKIT [39] to trim the alignments using the smart-gap option on default parameters.

To construct the phylogenies, we used IQ-TREE v.2.4.0 [40] with 1000 ultrafast bootstrap replicates. As additional branch support methods, we performed 1000 replicates of the SH-aLRT test (--alrt), likelihood-based bootstrap (--lbp), and applied the approximate Bayes test (--abayes). To detect possible reticulation events such as hybridization and introgression, we used the 90% matrix to generate a phylogenetic network in SplitsTree v.6.4.13 [41], using the NeighborNet algorithm based on p-distance calculations with 100 bootstrap replicates. We also applied the Phi Test (Φ Test) to statistically detect the presence of introgression.

Finally, to estimate divergence times within the group, we inferred a time-calibrated phylogeny using our 90% matrix and the LSD2 method (Least Squares Dating), as implemented in IQ-TREE v.2.4.0 [42]. To calibrate the phylogeny, we applied three calibration points: (1) the estimated date for the colonization of North America by *Astyanax* (based on Ornellas-García et al. [43]), which was used to constrain the tMRCA of the genus *Astyanax* to between 8 and 12 Mya; (2) the estimated divergence time between *A. lacustris* and *A. altiparanae* (based on Cunha et al. [44]), which was used to constrain the tMRCA between these two species to between 0.98 and 3.20 Mya; and (3) the estimated divergence time between *P. scabripinnis* and *P. paranae* from the Tietê, Grande, and Paranapanema river basins (based on Marreta [45]), which was used to constrain the tMRCA of *P. scabripinnis* and *P. paranae* to between 0.5 and 1.1 Mya.

### 2.4. Morphological and Morphometric Analyses

For the morphological characterization of the analyzed specimens, we performed meristic counts following Fink and Weitzman [46], except for the number of scales below the lateral line, for which we followed Bertaco and Lucena [47]. The chromatophore pattern on the anterior region of the body below the lateral line was interpreted according to Garutti and Langeani [48]. Morphometric measurements were taken according to Menezes and Weitzman [49], and as additional measurements, we included the postorbital length (POL), the head height (HH) and the caudal peduncle height (CPH2), both expressed as a percentage of body depth. In the taxonomy section, the number of individuals for each count is given in parentheses. When available, holotype, lectotype, or syntype counts are accompanied by an asterisk.

For the morphometric measurements, we used a total of 140 specimens, including both specimens analyzed firsthand, and photographs of specimens examined by Oliveira [14] as well as type specimens available on the website of the Natural History Museum of Denmark’s biological collections. To ensure standardization and avoid measurement biases resulting from different observers or photographic distortions, all specimens were digitized, and measurements were performed using Inkscape v.1.4.2 software. After the measurements, we selected a total of 16 morphometric characters (Table 2) that were consistently measurable across all specimens, and we added the number of scales along the longitudinal series to the dataset. This dataset was then used for statistical analyses, which were conducted in R v.4.4.2 [50] and R Studio v.2024.12.1+563 [51].

We divided our samples into five operational taxonomic units (OTUs) based on the congruence between morphological, cytogenetic, and phylogenetic groupings. After importing the data into the R environment, we tested for normality using the Shapiro–Wilk test and for homogeneity of variances using Levene’s test. Since these assumptions were violated for some traits, we used the Kruskal–Wallis test to assess the null hypothesis that the groups do not differ in their morphological data, followed by pairwise comparisons using Dunn’s test. We also conducted a Principal Component Analysis (PCA) to reduce data dimensionality and to identify which traits most strongly influenced the morphological variation among specimens. Finally, we employed a Random Forest analysis to determine which traits were most important for distinguishing among the OTUs and to evaluate how accurately specimens could be assigned to their respective groups.

## 3. Results

### 3.1. Morphotypes and Cytotypes of P. rivularis Sensu Lato

**Figure 2:**
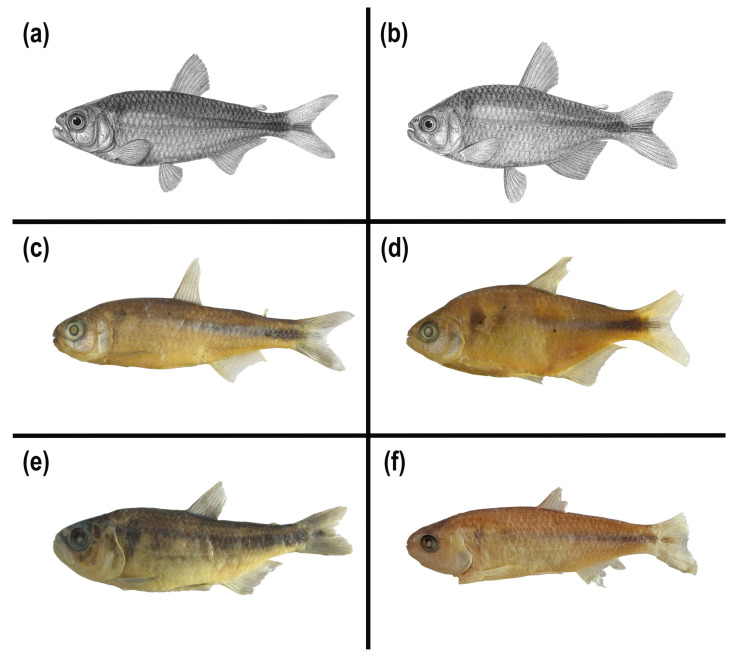
Morphotypes of *P. rivularis*, (**a**) morphotype 1 of Lütken—adapted from Lütken (1875); as reproduced by Alves & Pompeu [5], (**b**) morphotype 2 of Lütken—adapted from Lütken (1875); as reproduced by Alves & Pompeu [5], (**c**) morphotype 1—das Velhas River, 69.5 mm; (**d**) morphotype 2—Lage stream, 53 mm; (**e**) morphotype 3—Rasga Canga Waterfall, 77 mm; (**f**) morphotype 4—Bonito stream, 73 mm.

In this study, we identified at least four different morphotypes of *Psalidodon rivularis* sensu lato, two of which correspond to M1 and M2 illustrated by Lütken [5] (Figure 2a,b). These morphotypes are distributed across 17 tributaries of six major rivers (São Francisco, das Velhas, Abaeté, Indaiá, Borrachudo, and Paracatu) within the Upper and Middle São Francisco River hydrographic mesoregions, with several cases of sympatry involving at most two morphotypes (Figure 1). The morphotypes can be mainly distinguished based on body height, the number of scales along the longitudinal series, chromatophore patterns on the anterior region of the body below the lateral line, and chromosome number.

**Figure 3:**
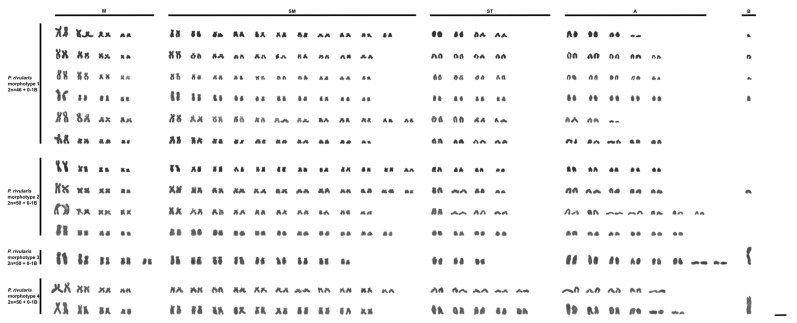
Karyotypes of *P. rivularis* separated by the identified morphotypes. From top to bottom: Lage stream (2*n* = 46 + B), Tiros stream (2*n* = 46 + B), Bonito stream (2*n* = 46 + B), Borrachudo River (2*n* = 46 + B), Funchal River (2*n* = 46), Crico stream (2*n* = 46), Lage stream (2*n* = 50), Lage stream (2*n* = 50 + B), Funchal River (2*n* = 50), Cipó River (2*n* = 50), Rasga Canga Waterfall from Serra da Canastra (2*n* = 50 + B), reservoir lagoon of the Usina do Abaeté dam (2*n* = 50), and Bonito stream (2*n* = 50 + B). Scale bar: 5 µm. M: metacentric chromosomes, SM: submetacentric, ST: subtelocentric, A: acrocentric and Bs: supernumerary chromosome.

Morphotype 1 (M1) corresponds to Figure 13 on Plate V of Lütken’s work [5] (Figure 2a,c) and includes the lectotype of “*T.*” *rivularis* deposited in the fish collection of the Natural History Museum of Denmark, as well as other paralectotypes. This morphotype is characterized by a shallower body, not exceeding 33% of the standard length, 37–39 scales along the longitudinal series, and a reticulated pattern of chromatophores on the anterior region of the body. All karyotyped specimens of this morphotype exhibited 2*n* = 46 chromosomes, usually presenting one or rarely two acrocentric supernumerary chromosomes with intercellular variation (Figure 3).

Morphotype 2 (M2) corresponds to Figure 14 on Plate V of Lütken’s work (1875) (Figure 2b,d), and includes paralectotypes of “*T.*” *rivularis*, as well as syntypes of *H. santae* and types of *A. turmalinensis*. This morphotype is characterized by a relatively deeper body than all others, almost always exceeding 33% of the standard length, 33–36 scales along the longitudinal series, and a reticulated pattern of chromatophores on the anterior region of the body. All karyotyped specimens of this morphotype exhibited 2*n* = 50 chromosomes, with a single specimen from the Lage stream (Abaeté River sub-basin) presenting a small acrocentric supernumerary chromosome (Figure 3).

The remaining two morphotypes are not represented in any of the type series of *P. rivularis*, *H. santae*, or *A. turmalinensis*. Morphotype 3 (M3, Figure 2e) corresponds to specimens collected from three localities within Serra da Canastra National Park, and is characterized by a shallower body, rarely exceeding 33% of the standard length, 37–39 scales along the longitudinal series, and a dispersed pattern of chromatophores on the anterior region of the body. All karyotyped specimens of this morphotype exhibited 2*n* = 50 chromosomes, with some carrying a large metacentric supernumerary chromosome (Figure 3).

Finally, morphotype 4 (M4, Figure 2f) was observed only in four tributaries of three rivers west of the São Francisco River (Abaeté, Borrachudo, and Paracatu). This morphotype is characterized by a shallower body, less than 33% of the standard length, 33–36 scales along the longitudinal series, and a reticulated pattern of chromatophores on the anterior region of the body. All karyotyped specimens of this morphotype exhibited 2*n* = 50 chromosomes, with some carrying a large metacentric supernumerary chromosome (Figure 3). The assembled karyotypes are shown in Figure 3 and summarized in Table 3.

### 3.2. Genomic Analysis

All assembled mitochondrial genomes ranged between 16,700 and 16,900 bp and had a GC content of 43%, except for the mitochondrial genome of *P. rivularis* M2 collected at the confluence of the Mascates and Bocaina rivers in the Serra do Cipó National Park, which had 17,086 bp and a GC content of 42%. Most of the variation in mitogenome size resides in the control region, since excluding it the mitogenomes ranged from 15,673 to 15,677 bp. Furthermore, all mitogenomes exhibited the same number of genes, composition, and organization typical of most vertebrate mitochondrial genomes, including teleosts, with: 13 protein-coding genes (PCGs), with only *ND6* found on the light strand; two rRNAs (*12S rRNA* and *16S rRNA*) on the heavy strand; 22 tRNAs, eight of which are located on the light strand; and the mitochondrial control region [52]. Details on the size, start and end position of each gene and the mitogenome control region can be found in supplementary material S5.

**Figure 4:**
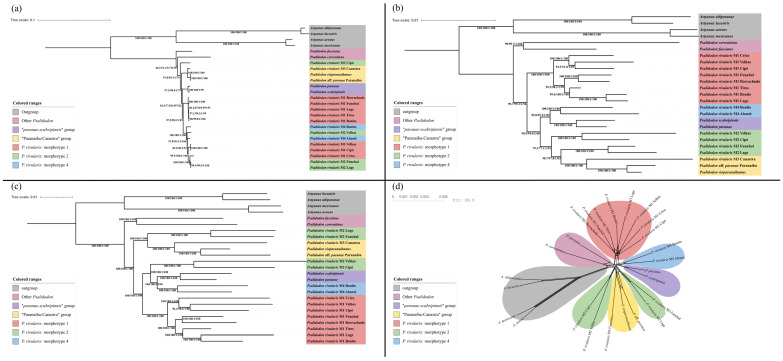
(**a**) Maximum Likelihood tree constructed from 15 mtDNA genes, totaling 14,027 bp in the final alignment. (**b**) Maximum Likelihood tree constructed from 51 orthologous PCGs from the 100% matrix, final alignment: 85,827 bp. (**c**) Maximum Likelihood tree constructed from 568 orthologous PCGs from the 90% matrix, final alignment: 860,113 bp. (**d**) Neighbor-Net based on the 860,113 bp alignment from the 90% matrix. Elongated rectangular boxes indicate relationships with little conflict in the data, while nearly square boxes with short branch lengths highlight clades with low support and high conflict. Support values at the nodes in (**a**–**c**) represent, respectively: SH-aLRT support (%), local bootstrap support (%), aBayes support, and ultrafast bootstrap support (%).

After alignment, partitioning, and trimming of the mitochondrial genes, we obtained an alignment with 14,027 bp. Our Maximum Likelihood phylogeny in IQ-TREE yielded a final score of −41,161.857758 and, overall, showed congruence and high support values across different support methods (Figure 4a). We recovered a clade containing all *Psalidodon* species related to the *P. scabripinnis* complex (i.e., excluding *P. fasciatus* and *P. correntinus*). Most mitogenomes of *P. rivularis* sensu lato clustered together in a single clade, sister group to a clade containing *P. paranae* and *P. scabripinnis*, which we refer to as the “*paranae–scabripinnis* group” (purple clade). Two samples, however, showed discordant mitochondrial placements (see below), rendering the group polyphyletic.

Within the previously *P. rivularis* clade related to *paranae-scabripinnis* group, we observed two well-structured subclades. The first consisted exclusively of M1 individuals collected from tributaries in the western Upper São Francisco mesoregion (Abaeté, Indaiá, and Borrachudo rivers). The second comprised the remaining M1 individuals from the Crico stream (a tributary of the Paracatu River in the Middle São Francisco), as well as from tributaries of the Cipó and das Velhas rivers in the eastern Upper São Francisco. This second clade also included individuals of M2 and M4, with no apparent structure among them, which makes morphotypes M1, M2 and M4 polyphyletic in the mitochondrial tree.

Two mitochondrial genomes fell outside this clade: *P. rivularis* M2 from Serra do Cipó National Park, retrieved as sister group to the remaining lineages of the former *P. scabripinnis* complex; and *P. rivularis* M3 from Serra da Canastra National Park, retrieved as sister group to *P. rioparanaibanus* + *P.* aff. *paranae* from the Paranaíba River, forming the clade we refer to as the “Paranaíba-Canastra Group” (yellow clade).

Our 100% matrix contains 51 orthologous genes shared among all libraries, with a final alignment length of 85,827 bp, which resulted in a final likelihood score of −201,728.8494 in the maximum likelihood phylogeny (Figure 4b). In this phylogeny, we also recovered a clade comprising all species related to the *P. scabripinnis* complex, as well as the “*paranae-scabripinnis*” and “Paranaíba-Canastra” groups, all with high support values. Unlike the mtDNA phylogeny, we obtained two major clades within the *P. scabripinnis* complex, one of which is composed entirely of *P. rivularis* M1 specimens (red clade). These are divided into two independent clades, consistent with the mtDNA results: one containing specimen from the western part of the Upper São Francisco mesoregion and the other including specimens from the Middle São Francisco and eastern Upper São Francisco.

Within the other clade of the *P. scabripinnis* complex, we recovered the two *P. rivularis* M4 specimens as a monophyletic group (blue clade), sister group to the “*paranae-scabripinnis*” group (purple clade). The four *P. rivularis* M2 specimens (in green), in turn, formed a paraphyletic assemblage in relation to the “Paranaíba-Canastra” group (yellow clade), and were organized into two clades: one comprising samples from the eastern part of the Upper São Francisco mesoregion (headwaters of the das Velhas River and the Cipó River), and another with samples from the western part of the Upper São Francisco mesoregion (Lage stream and Funchal River).

Our 90% matrix contains a total of 568 orthologous PCGs recovered in at least 23 out of the 25 libraries, yielding a final alignment of 860,113 bp and a final maximum likelihood score of −2,203,710.6475 (Figure 4c). All branch supports reached the maximum value across all applied methods, except for a single grouping containing *P. rivularis* M1 specimens from the Cipó and das Velhas rivers. Overall, the phylogeny topology was very similar to that of the 100% matrix, but with *P. rivularis* M2 (in green) recovered as a polyphyletic group. In this phylogeny, the *P. rivularis* M2 specimens from the eastern part of the Upper São Francisco mesoregion appeared as a sister group to the *P. rivularis* M4 clade + “*paranae-scabripinnis*” group, and the *P. rivularis* M1 clade “swapped” positions with the clade containing *P. rivularis* M2 from the western Upper São Francisco + “Paranaíba-Canastra” clade.

The Phi test rejected the null hypothesis of absence of recombination (*p* = 0.0) in the 90% matrix, and the phylogenetic network presented a reticulate topology with 62 branches (Figure 4d), suggesting hybridization and introgression processes in the evolutionary history of the group. Despite the large number of reticulations, the phylogenetic network tends to agree with the groupings of the clades *P. rivularis* M1 (bootstrap = 100), *P. rivularis* M4 (bootstrap = 100), “*paranae-scabripinnis*” group (bootstrap = 100), and “Paranaíba-Canastra” group (bootstrap = 96). In contrast, a grouping uniting *P. rivularis* M2 from the east and west of the Upper São Francisco River has low support (bootstrap = 61).

According to our time-calibrated phylogeny, the tMRCA of *Astyanax* and *Psalidodon*, i.e., the root of the tree, was estimated at 9.3 Mya (95% CI: 8.7–11.4 Mya). The tMRCA of all *Psalidodon* included in the analysis was estimated at 5.8 Mya (95% CI: 5.1–7.1 Mya), and that of the clade containing all *Psalidodon*, except *P. fasciatus* and *P. correntinus*, at 5.6 Mya (95% CI: 4.9–6.2 Mya).

Regarding the different groups containing *P. rivularis*, the tMRCA of the Paranaíba–Canastra clade was estimated at 4.0 Mya (95% CI: 3.1–5.2 Mya), and its divergence from *P. rivularis* M2 from the western Upper São Francisco River at 5.3 Mya (95% CI: 4.7–6.4 Mya). The tMRCA of the “*paranae–scabripinnis*” clade and *P. rivularis* M4 was estimated at 3.5 Mya (95% CI: 3.0–4.3 Mya), and the divergence between these groups and *P. rivularis* M2 from the eastern Upper São Francisco River at 4.6 Mya (95% CI: 4.0–5.6 Mya). Finally, the tMRCA of *P. rivularis* M1 was estimated at 3.9 Mya (95% CI: 3.3–5.0 Mya), and its divergence from the other groups at least 5.1 Mya (95% CI: 4.4–6.1 Mya). Our time-calibrated phylogeny is available in Supplementary Material S6.

### 3.3. Morphological Analyses

For the statistical morphological analyses, we separated the specimens *of Psalidodon rivularis* sensu lato into five groups, corresponding to the four identified morphotypes and, based on the results of the phylogenomic analyses, further dividing the specimens of M2 into two groups: west of the São Francisco River (Lage stream and Funchal River) and east of the São Francisco River (type series of *H. santae*, *A. turmalinensis*, and *P. rivularis* specimens from Serra do Cipó National Park and the das Velhas River). The Kruskal–Wallis test rejected the null hypothesis (*p* < 0.05) for all 16 morphometric traits and for the number of scales along the lateral series, indicating significant differences in these traits among the five OTUs of *P. rivularis* sensu lato. The pairwise comparison results of Dunn’s test for each of the 17 traits are summarized in Table 4.

**Figure 5:**
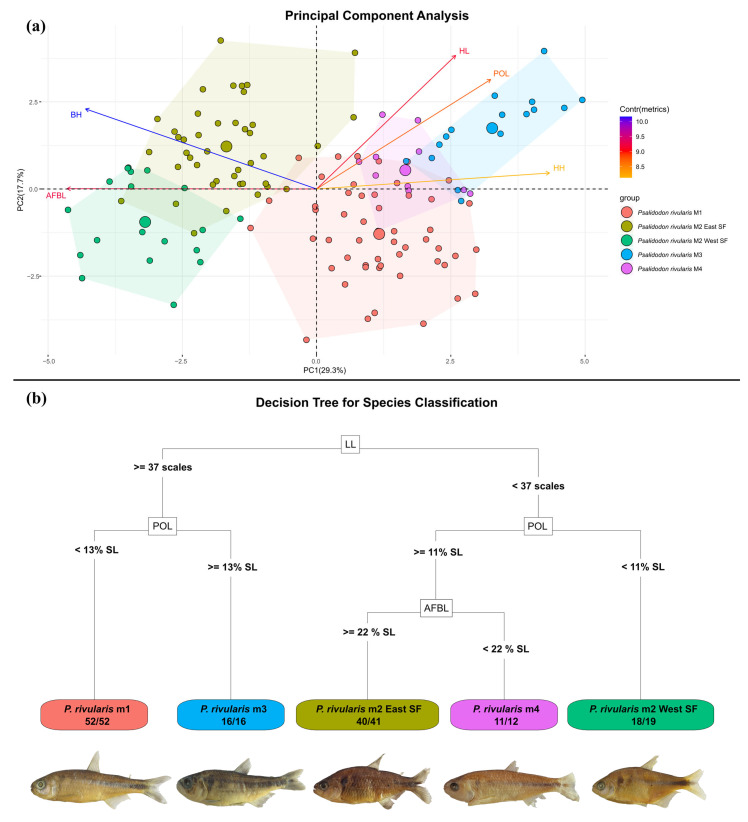
(**a**) Principal Component Analysis of the 16 morphometric characteristics used plus the number of scales along the lateral series. The five characteristics with the greatest contribution to the two principal components are plotted. (**b**) Decision Tree for Classification of morphotypes into species. The values within each balloon indicate the number of specimens correctly classified in each group and the total number of specimens classified in the group.

The ordination of specimens in the principal component analysis (PCA) revealed a clear separation of M2 (both eastern and western São Francisco specimens) from the other *P. rivularis* morphotypes (M1, M3, and M4) along PC1, which accounted for 29.3% of the variation (Figure 5a). Along with PC2, which explained 17.7% of the variation, we observed mainly the separation between the two groups of M2 and between specimens of M1 and M3, with M4 occupying an intermediate position between them. When plotting the five characteristics with the highest contribution to the two main axes, we observed that specimens of M2 are primarily distinguished by having greater body height (BH) and anal fin base length (AFBL). In contrast, the separation between the two groups of M2, as well as among M1, M3, and M4, is mainly due to head length (HL), head height (HH), and postorbital length (POL), with M3 specimens showing high values for these traits.

Our Random Forest model achieved an overall accuracy of 97.86% (CI: 93.87–99.56%), and the hypothesis test comparing the model’s accuracy to the no-information rate (NIR) rejected the null hypothesis that the model is not statistically better than random classification (*p* = 2.2 × 10^−16^). The decision tree generated used three main traits to classify the specimens (Figure 5b): number of scales along the lateral series (LL), postorbital length (POL), and anal fin base length (AFBL). The resulting confusion matrix showed sensitivity and specificity values above 94% and 99%, respectively, for all classifications (Table 5).

### 3.4. Taxonomy

Considering the genera currently included in Acestrorhamphidae Melo et al. 2024 that contain species historically assigned to the genus *Astyanax* Baird and Girard 1854 (sensu lato), *H. santae* (Eigenmann 1907) and *A. turmalinensis* Triques, Vono and Caiafa 2003, as well as *P. rivularis* (Lütken 1875), can be assigned to the genus *Psalidodon* Eigenmann 1911, rather than to *Astyanax* Baird & Girard 1854 (sensu stricto), *Hemigrammus* Gill 1858 (sensu stricto), *Deuterodon* Eigenmann 1907, *Hyphessobrycon* Durbin 1908 (sensu stricto), *Megalamphodus* Eigenmann 1915, *Jupiaba* Zanata 1997, *Andromakhe* Terán, Benitez and Mirande 2020, or *Makunaima* Terán, Benitez and Mirande 2020, based on the following combination of characters: absence of circuli on the posterior margin of the scales (vs. present in *Astyanax* and *Jupiaba*), presence of a black spot on the caudal peduncle (vs. absent in *Hemigrammus*), anterior laterosensory pore to the dilator fossa oriented lateroventrally (vs. dorsomedially oriented in *Deuterodon*), absence of the dorsal expansion of the rhinosphenoid between the olfactory nerves (vs. present in *Deuterodon*, *Jupiaba*, and *Makunaima*), numerous and small hooks per ray on the anal fin of males (vs. a pair of large hooks per ray on the anal fin in *Hyphessobrycon*, and absence of hooks in *Jupiaba*), absence of a conspicuous black blotch on the dorsal fin (vs. present in *Megalamphodus*), origin of the anal fin posterior to the vertical through the last dorsal-fin ray (vs. anterior in *Andromakhe*), and presence of a longitudinal black stripe (vs. absent in *Makunaima*).

#### 3.4.1. ***Psalidodon rivularis* (Lütken 1875)**

**Figure 6:**
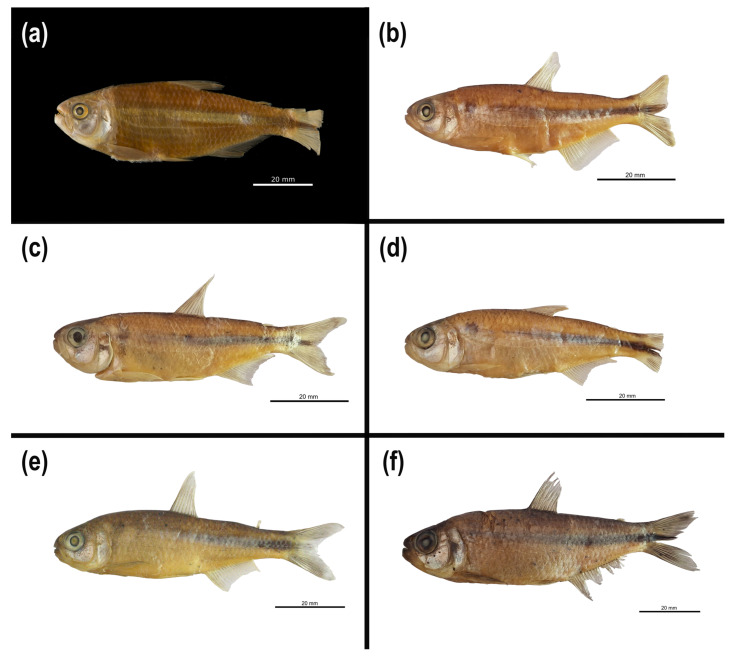
*P. rivularis*, (**a**) lectotype, ZMUC P241289, 80.7 mm SL, das Velhas River, (**b**) LaGEEvo 34-4770, 59.0 mm SL, Borrachudo River, (**c**) LaGEEvo 43-4326, 63.0 mm SL, Crico stream, Paracatu River drainage, (**d**) LaGEEvo 30-4510, 63.5 mm SL, Lage stream, Abaeté River drainage, (**e**) LaGEEvo 33-5128, 69.5 mm SL, das Velhas River and (**f**) LaGEEvo 48-5152, 84.0 mm SL, confluence of the Mascates and Bocaina rivers, Cipó River drainage. (**a**) available at: https://collections.snm.ku.dk/en/object/NHMD1634879 (accessed on 17 September 2024), under license: http://creativecommons.org/licenses/by/4.0/ (accessed on 17 September 2024).

(Figure 6a–f; Table 6)

*Tetragonopterus rivularis* Lütken 1875:107109, board V, Figure 13 (in part; lectotype and paralectotypes: ZMUC, MNHN 0000-9582, NMW 57707, SMNS 2046, ZMB 9199, USNM 44960, type locality: das Velhas River and its tributaries, Minas Gerais state, Brazil) [5]— Bertin and Estève 1948:21 (catalog of fish types from the Muséum national d’Histoire Naturelle) [53]— Nielsen 1974:46 (catalog of fish types from Zoological Museum of Copenhagen) [54]— Fricke 1995:9 (catalog of fish types from Staatliches Museum für Naturkunde in Stuttgart) [55].

*Astyanax scabripinnis rivularis*— Eigenmann 1910: 433 (transfer to *Astyanax* as a subspecies of *Astyanax scabripinnis*) [56]— Moreira-Filho and Bertollo 1991:331-357 (citation as a valid subspecies in the *A. scabripinnis complex*) [3].

*Astyanax rivularis*— Casatti and Castro 1998:232 (valid species in the genus *Astyanax*) [57]— Buckup in Reis et al., 2003:112 (list of species) [58]— Bertaco and Lucena 2006: 58 (citation as a valid species in the *A. scabripinnis* complex) [47]— Ingenito and Duboc 2014: 282 (Citation in the *A. scabripinnis* complex) [59]— Pasa et al. 2019: 307-314 (distribution in the Upper São Francisco River) [60]— Silva et al. 2020:6 (list of species, expansion of distribution to the state of Bahia, Brazil) [61].

*Psalidodon rivularis*— Terán, Benitez and Mirande 2020:11 (transfer to *Psalidodon*) [2]— Rodrigues-Oliveira et al. 2023 (distribution in upper and middle São Francisco River) [62]— Quintela, Teixeira and Pompeu et al. 2024:6 (ichthyofauna Lagoa Santa, state of Minas Gerais, Brazil) [63].

**Diagnosis.** *Psalidodon rivularis* differs from most of its congeners by the following combination of characters: 14–21 branched anal-fin rays (vs. 22 or more), 37–39 scales in the lateral series (vs. 36 or fewer, or 40 or more), and body depth equal to or less than 33.1% of standard length (vs. greater than 33.1% SL). Among the remaining species, *P. rivularis* differs from *P. brachypterygium*, *P. cremnobates*, *P. ojiara*, *P. paranae*, and *P. varzeae* by the presence of a reticulated chromatophore pattern on the anterior region of the body below the lateral line (vs. a diffuse pattern); from *P. dissimilis*, *P. hermosus*, *P. jequitinhonhae*, *P. laticeps*, *P. paranae*, *P. scabripinnis*, *P. serratus*, and *P. varzeae* by having two humeral spots (vs. one); from *P. gymnodontus* and *P. varzeae* by the presence of hooks on the anal and pelvic fins of males (vs. hooks absent); from *P. hermosus*, *P. leonidas*, *P. ojiara*, and *P. troya* by the absence of hooks on the caudal fin of males (vs. hooks present); and from *P. laticeps*, *P. scabripinnis*, *P. serratus*, and *P. troya* by having a vertically elongate humeral spot (vs. a horizontally elongate humeral spot with an anterior vertical projection). *Psalidodon rivularis* further differs from *P. dissimilis* by the upper jaw length (29.5–47.4% HL vs. 24.3–29.2%); from *P. eigenmanniorum* and *P. jequitinhonhae* by having, in general, a shorter anal-fin base (16.4–24.2% SL vs. 23.7–35.6% in *P. eigenmanniorum* and 23.6–32.0% in *P. jequitinhonhae*); from *P. eigenmanniorum* by the pectoral-fin tip not reaching the pelvic-fin origin (vs. reaching) and the pelvic-fin tip not reaching the anal-fin origin (vs. reaching); and from *P. xiru* by having tricuspid teeth in the outer premaxillary row (vs. pentacuspid teeth).

**Description.** Morphometric data are available in Table 6. Body compressed, greatest body depth generally located at the vertical passing through the middle of the pectoral fin or rarely located at the vertical near the origin of the dorsal fin (in some individuals with body depth greater than 30.50% of standard length). Dorsal profile of the head slightly convex from the tip of the upper lip to the vertical anterior to the nostril; usually convex, but sometimes straight or slightly concave from this point to the supraoccipital process; convex from this point to the base of the last dorsal-fin ray; slightly convex, sometimes straight, from the dorsal fin to the adipose fin; slightly concave between the adipose fin and the base of the uppermost caudal-fin ray. Ventral profile from the tip of the snout to the base of the pelvic fin convex; from the pelvic fin to the base of the first anal-fin ray continuously convex or straight; anal-fin base straight; and slightly concave between the last anal-fin ray and the base of the lowermost caudal-fin ray.

Terminal mouth with lower jaw slightly projecting beyond upper jaw. Premaxilla with two tooth series: outer series with 3(3) or 4(13*) tricuspid teeth and inner series with 4(6) or 5(9*) teeth bearing 4–7 cusps. Maxilla with 1(5) or 2(8*) small tricuspid or pentacuspid teeth. Dentary teeth abruptly decreasing in size posteriorly, with 4(6) or 5(10*) large teeth bearing 5–6 cusps followed by 4–9 smaller teeth. In all teeth, the central cusps are larger than the lateral cusps. Maxilla extending posteriorly beyond the vertical through the anterior margin of the orbit.

Dorsal fin with ii+9 rays, first unbranched ray about half the length of the second. Pectoral fin with i+11(10), 12(10*), 13(14), or 14(3) rays, origin near the vertical through the middle of the opercular bone; when adpressed against the body, never reaching the pelvic fin in larger individuals. Pelvic fin with i+6(2) or 7(30) rays, origin anterior to the vertical through the last dorsal-fin ray; when adpressed against the body, never reaching the anal fin in larger individuals. Anal fin with iv+14(3), 15(1), 16(5), 17(15), 18(19), 19(28*), 20(21), or 21(1) rays; mature males with numerous small hooks per ray on the anal fin. Caudal fin forked, with i+17+i rays and lobes of similar size. Adipose fin present.

Cycloid scales, circuli absent on the posterior margin of scales; 4–17 radii on scales, generally more numerous in larger individuals. Lateral line variable, complete (109), incomplete (2), or interrupted (22*). Lateral series with 37(56*), 38(60), or 39(32) scales. Perforated scales along lateral series with 32(4), 33(3), 34(1), 35(6*), 36(3), 37(47), 38(49), or 39(25) scales. Transverse scale series with 5(127) or 6(27*) scales above and 4(109) or 5(27*) scales below the lateral line. Predorsal scale series with 11(6), 12(24*), or 13(8) scales. Circumpeduncular scale series with 11(2), 12(5), 13(17), 14(12), or 15(1) scales. Anal-fin base scale series with 3(5), 4(15), 5(7), 6(3), or 7(3) scales.

**Coloration in alcohol.** Dorsal region of the body, head, and tip of the snout dark brown. Lateral and ventral regions of the body yellowish-brown or slightly silvery. Infraorbital and opercular regions are silvery, with a dark patch of chromatophores on the opercle. Reticulated pattern of chromatophores on the anterior region of the body below the lateral line, that is, restricted to the posterior margin of the scales. Scattered pattern of chromatophores between the lateral line and the base of the anal fin. Conspicuous humeral blotch extending 2–3 scales above the lateral line and 1–2 scales below, with the upper margin wider than the lower margin. Some specimens have a second diffuse humeral blotch after the first; its absence in others may be due to the preservation process. Regions anterior and posterior to the first humeral blotch are pale, with some specimens showing a reticulated pattern of chromatophores after the blotch. Lateral stripe extending from the second humeral blotch to the median rays of the caudal fin, silvery in its anterior portion and becoming darker toward the caudal peduncle, where it extends dorsoventrally forming a distinct blotch on the margin of the caudal peduncle. Rayed fins hyaline, generally with few chromatophores along the margins of the rays. Adipose fin hyaline with a scattered pattern of chromatophores.

**Live coloration.** Dorsal region of the body and head dark, brownish green on the back, becoming olive-green toward the flanks. Area around the lateral line silvery, turning whitish on the belly. Paired fins whitish. Dorsal fin white or yellowish. Adipose fin yellowish. Anal fin whitish, sometimes with the margin near the body yellowish to reddish. Caudal fin with broad reddish and yellowish margins surrounding the dark lateral stripe.

**Distribution.** *Psalidodon rivularis* sensu stricto can be found in the upper and middle São Francisco River basins, from its easternmost portion in the Cipó and das Velhas rivers to its westernmost portion in the Abaeté and Paracatu rivers. Among other rivers where this species occurs, noteworthy are the sub-basins of the Indaiá and Borrachudo rivers, as well as smaller tributaries that drain directly into the São Francisco River (Figure 1).

**Sexual dimorphism.** Reproductive males bear hooks on the anal, pectoral and pelvic fins, from the last unbranched ray to the 15th branched ray of the anal fin, from the last unbranched ray to the 8th branched ray of the anal fin, and from the 3rd to the 5th branched ray of the pelvic fin (see Oliveira [14]).

**Remarks.** Here we define *Psalidodon rivularis* sensu stricto as only the M1 individuals of *P. rivularis* sensu lato (Figure 4, red clade). This interpretation is based on the confirmation of two distinct species historically identified under the name *Tetragonopterus rivularis* in the type series analyzed by Lütken [5], or later as *Psalidodon*/“*Astyanax*” *rivularis* in other ichthyological collections (eg., LaGEEvo UFV CRP). We restrict *Psalidodon rivularis* sensu stricto as all specimens belonging to the same morphotype as the lectotype of *Tetragonopterus rivularis* (NHMD1634879), whose photograph and radiograph are available on the Natural History Museum of Denmark collections website (https://collections.snm.ku.dk/en (accessed on 17 September 2024)), and in Figure 13 of plate V of Lütken’s work Velhas-Flodens Fiske [5].

This narrower definition reduces the morphological variability attributed to the species in previous works. Among the affected characteristics, the most notable are the number of scales along the lateral series and morphometric proportions, particularly body height. Lütken [5] described *Tetragonopterus rivularis* as having 33–38 scales along the lateral series, while other studies expanded this range to 32–40 [60,64]. Under our definition, *Psalidodon rivularis* sensu stricto presents 37–39 scales along the lateral series. Similarly, based on Lütken’s [5] notes, it is possible to infer that the specimens he analyzed had a body height ranging from 31.58% to 37.50% of the standard length. Other studies broaden this range in *Psalidodon rivularis* to 22.06–46.00% [47,60]. Here, we define *Psalidodon rivularis* as individuals with proportionally shallower bodies, with body height ranging from 25.30% to 33.10% of the standard length.

**Material Examined.** 154 specimens (4 from photographs), all from the state of Minas Gerais, Brazil. **Types: São Francisco River Basin. Sub-basin of the Velhas River:** ZMUC P241289 (photograph), lectotype of *Tetragonopterus rivularis*, 1 specimen, standard length: 80.7 mm, Lagoa Santa municipality–MG, J. T. Reinhardt, 1847–1870. USNM 44960 (photograph), paralectotypes of *Tetragonopterus rivularis*, 2 specimens, standard length: 36.6–67.8 mm, Lagoa Santa municipality–MG, J. T. Reinhardt, 1847–1870. **Non-types. São Francisco River Basin. Sub-basin of the Abaeté River:** LaGEEvo-30, 12 of 13 specimens, standard length: 45–63.5 mm, Arapuá municipality–MG, Lage stream, 19°1′25.18″ S 46°6′18.74″ W, I.H.R. Oliveira, P. M. de Assis, T. da S. Ramos, 5 November 2022. LaGEEvo-41, 17 of 31 specimens, standard length: 48–79.5 mm, Arapuá municipality–MG, Lage stream, 19°1′25.18″ S 46°6′18.74″ W, P. Penteado, D. Reis, Denis, Paloma, Wanessa, 23 July 2010. LaGEEvo-56, 14 of 18 specimens, standard length: 48–69 mm, Tiros municipality–MG, Tiros stream, 18°56′34.08″ S 45°56′18.20″ W, P. Penteado, Denis, Gabriel, Rafael, 20 July 2010. LaGEEvo-74, 11 specimens, standard length: 42–91 mm, São Gotardo municipality–MG, Confusão stream, 19°20′21.89” S 46°6′21.38” W, I. H. R. Oliveira, P. M. de Assis, W. Cléber, 11 November 2024. LaGEEvo-75, 4 specimens, standard length: 56–87.5 mm, Tiros municipality–MG, Espinha stream, 19°2′47.18″ S 46°1′12.93″ W, Campos, M. A. da Silva, S. V. Resende, R. Pasa, 24 July 2016. **Sub-basin of the Velhas River:** MCZ 20874 (photograph), 1 of 3 specimens, standard length: 67.61 mm, Lagoa Santa–MG, 19°27′26″ S 44°14′30″ W, G. Sceva & Thayer Expedition, July 1865. LaGEEvo-33, 4 specimens, standard length: 46.0–69.5 mm, Ouro Preto municipality–MG, Velhas River, 20°20′38.4″ S 43°29′58.4″ W, I.H.R. Oliveira, I. B. da Silva, P. M. de Assis, L. G. P. Pimentel, R. Pasa, 15 September 2023. **Sub-basin of the Borrachudo River:** LaGEEvo-34, 3 specimens, standard length: 53–69 mm, Matutina municipality–MG, Borrachudo River, 19°13′02.6″ S 45°55′58.2″ W, I.H.R. Oliveira, P. M. de Assis, R. A. S. Soares, R. Pasa, 8 March 2023. LaGEEvo-40, 12 of 15 specimens, standard length: 64–86 mm, Tiros municipality–MG, Bonito stream, 18°48′44.7″ S 45°45′52.2″ W, P. Penteado, Denis, Gabriel, Rafael, 20 July 2010. **Sub-basin of the Indaiá River:** LaGEEvo-36, 4 specimens, standard length: 41–46 mm, São Gotardo municipality–MG, Funchal River, 19°24′9.54″ S 46°0′4.61″ W, I.H.R. Oliveira, I. B. da Silva, P. M. de Assis, L. G. P. Pimentel, 2 December 2023. LaGEEvo-37, 2 specimens, standard length: 47–56 mm, São Gotardo municipality–MG, Funchal River, 19°24′9.54″ S 46°0′4.61″ W, I.H.R. Oliveira, I. B. da Silva, L. Fainé, J. Godoy, 4 May 2019. LaGEEvo-59, 3 specimens, standard length: 50–65 mm, São Gotardo municipality–MG, Funchal River, 19°24′9.54″ S 46°0′4.61″ W, I.H.R. Oliveira, I. B. da Silva, G. Bork, L. Fernandes, V. Augusto, 12 May 2018. **Sub-basin of the Paracatu River:** LaGEEvo-43, 9 specimens, standard length: 37–63 mm, Presidente Olegário municipality–MG, Crico stream, 18°18′44.36″ S 46°5′44.46″ W, I. B. da Silva, M. L. C. B. de Campos, V. Augusto, S. V. Resende, 6 April 2019. **Sub-basin of the Cipó River:** LaGEEvo-44, 4 specimens, standard length: 43–52 mm, Serra do Cipó National Park–MG, Bandeirinhas Canyon, 19°25′8.33″ S 43°34′12.37″ W, M. L. C. B. de Campos, R. R. Rocha, S. V. Resende, F. Sassi, October 2017. LaGEEvo-46, 9 specimens, standard length: 42–59 mm, Serra do Cipó National Park–MG, Bandeirinhas stream, 19°24′32.65″ S 43°34′35.31″ W, M. L. C. B. de Campos, R. R. Rocha, S. V. Resende, F. Sassi, October 2017. LaGEEvo-48, 23 specimens, standard length: 47–84 mm, Serra do Cipó National Park–MG, confluence of Mascates and Bocaina Rivers, 19°20′49.68″ S 43°36′20.42″ W, I.H.R. Oliveira, I. B. da Silva, P. M. de Assis, L. G. P. Pimentel, R. A. S. Soares, G. F. da Fonseca, G. F. Matos, V. G. de Miranda, B. Alonso, 9 October 2023. LaGEEvo-60, 11 specimens, standard length: 64–99 mm, Serra do Cipó National Park–MG, Farofa Waterfall trail, 19°23′6.52″ S 43°35′12.28″ W, I.H.R. Oliveira, I. B. da Silva, P. M. de Assis, L. G. P. Pimentel, R. A. S. Soares, G. F. da Fonseca, G. F. Matos, V. G. de Miranda, B. Alonso, 10 October 2023. **São Francisco River:** LaGEEvo-63, 4 specimens, standard length: 50–81 mm, Três Marias–MG, Vereda Grande River, 18°19′18.62″ S 45°6′32.80″ W, R. de M. Alves; R. R. Rocha; M. A. da Silva; S. V. Resende, 18 Apr 2017. LaGEEvo-64, 5 specimens, standard length: 50–81 mm, Três Marias–MG, Vereda Grande River, 18°19′18.62″ S 45°6′32.80″ W, 2010–2012.

#### 3.4.2. **New Combinations: *Psalidodon santae* (Eigenmann, 1907) comb. nov.**

**Figure 7:**
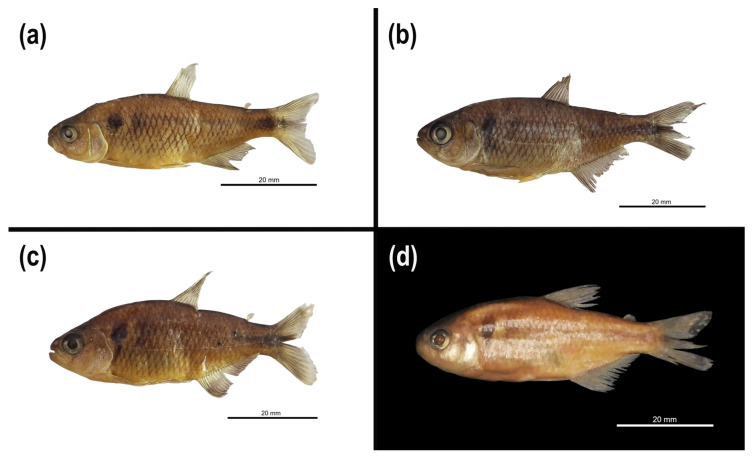
*P. santae*, (**a**) LaGEEvo 32-5129, 49.0 mm SL, source of the das Velhas River, (**b**) LaGEEvo 49-5173, 54.5 mm SL, confluence of the Mascates and Bocaina rivers, Cipó River drainage, (**c**) LaGEEvo 45-4064, 52 mm SL, Bandeirinhas Cânion, Cipó River drainage and (**d**) holotype of “*A. turmalinensis*”, DZUFMG 005, 48.2 mm SL, Divisão stream, Jequitinhonha River drainage.

(Figure 7a–d Table 7)

*Tetragonopterus rivularis* Lütken, 1875: 107-109, board V, Figure 14 (in part; paralectotypes: ZMUC, MNHN 0000-9582, NMW 57707, SMNS 2046, ZMB 9199, type locality: das Velhas River and its tributaries, Minas Gerais state, Brazil) [5].

*Hemigrammus santae* Eigenmann 1907: 16-17, (syntypes: USNM 55652, type locality: municipality of Lagoa Santa, Minas Gerais state, Brazil) [6]— Vari and Howe 1991:25 (species catalog of the National Museum of Natural History, Smithsonian Institution) [65].

*Hyphessobrycon santae*— Eigenmann 1910:437 (Transfer to the genus *Hyphessobrycon*) [56]— Lima and Malabarba in Reis et al. 2003:140 (list of species) [66]— Silva et al. 2020:6 (list of species, expansion of distribution to the state of Bahia, Brazil) [61].

*Astyanax turmalinensis* Triques, Vono and Caiafa 2003: 145–150, Figure 1 (Holotype: DZUFMG: 005; Paratypes: DZUFMG: 006-009, type locality: Divisão stream, tributary of the right bank of the Jequitinhonha River, village of Peixe Crú, municipality of Turmalina, Minas Gerais state, Brazil, 17°07′ S 42°57′ W.) [7]— Zanata and Camelier 2009: 37 (inclusion in the *A. scabripinnis* complex) [67]— Triques and Queiroz 2010: 400–401, Figure 1 (expansion of distribution to the São Francisco and Doce river basins) [8]— Ingenito and Duboc 2014: 282 (Citation in the *A. scabripinnis* complex) [59]— Vieira-Guimaraes et al. 2024:37 (list of species) [68]. **[Syn. nov.]**.

**Diagnosis.** *Psalidodon santae* differs from most of its congeners by the following combination of characters: 16–22 branched anal-fin rays (vs. 23 or more, or 14 or fewer), 33–36 scales in the lateral series (vs. 37 or more), and hooks on the anal and pelvic fins of males (vs. absence on either of these fins or presence on the dorsal and caudal fins). Among the remaining species, *P. santae* differs from *P. anisitsi*, *P. balbus*, *P. dissimilis*, *P. jequitinhonhae*, *P. laticeps*, *P. minor*, *P. scabripinnis*, and *P. serratus* by having two humeral spots (vs. one); from *P. bifasciatus*, *P. laticeps*, *P. scabripinnis*, and *P. serratus* by possessing a vertically elongate humeral spot (vs. a horizontally elongate humeral spot with an anterior vertical projection); from *P. brachypterygium*, *P. cremnobates*, *P. goyanensis*, *P. powelli*, and *P. rioparanaibanus* by the presence of a reticulated chromatophore pattern on the anterior region of the body below the lateral line (vs. a diffuse pattern); and from *P. eigenmanniorum*, *P. endy*, and *P. pampa* by the pectoral-fin tip not reaching the pelvic-fin origin (vs. reaching) and the pelvic-fin tip not reaching the anal-fin origin (vs. reaching). *Psalidodon santae* further differs from *P. balbus* and *P. bockmanni* by having body depth less than 39% of standard length (vs. greater than 40%); from *P. biotae* by having an anal-fin base length less than 29% of standard length (vs. greater than 29%); from *P. dissensus* by having tri- or pentacuspid maxillary teeth (vs. heptacuspid teeth); and from *P. pessalii* by the presence of an adipose fin (vs. adipose fin absent). Finally, *P. santae* differs from *P. rivularis* by having 33–36 scales in the lateral series (vs. 37–39) and 2*n* = 50 chromosomes (vs. 2*n* = 46).

**Description.** Morphometric data are available in Table 7. Body compressed, greatest body depth located on the vertical near the origin of the pelvic fin. Dorsal profile of head slightly convex between the tip of the upper lip and the vertical anterior to the nostril; slightly convex from this point to the vertical situated near the middle of the eye, and then straight or slightly concave from this point to the supraoccipital process. Convex profile from the supraoccipital process to the base of the last dorsal-fin ray; slightly convex from the dorsal to the adipose fin, and slightly concave between the adipose fin and the base of the uppermost caudal-fin ray. Ventral profile from tip of snout to pelvic-fin base convex; between pelvic fin and base of first anal-fin ray continuously convex or straight; anal-fin base straight; and slightly concave between adipose fin and base of the lowermost caudal-fin ray.

Mouth terminal, lower jaw slightly projecting beyond upper jaw. Premaxilla with two series of teeth, the outer with 3(5*), 4(6*), or 5(1) tricuspid teeth and the inner with 4(8**) or 5(4) teeth bearing 3–7 cusps. Maxilla with 1(2), 2(7*), or 3(3*) small tricuspid or pentacuspid teeth. Dentary with teeth decreasing abruptly in size, 4(5) or 5(7**) large teeth with 4–5 cusps followed by 3–8 smaller teeth. In all teeth, the central cusps are larger than the lateral cusps. Posterior end of maxilla extending beyond vertical through anterior margin of orbit.

Dorsal fin with ii+9 rays, first unbranched ray half the length of the second. Pectoral fin with i+11(13**), 12(10), or 13(6) rays, its origin near the vertical through the middle of the opercle; when adpressed against the body, it never reaches the pelvic fin in larger individuals. Pelvic fin with i+6(2*) or 7(20*) rays, its origin anterior to the vertical through the last dorsal-fin ray; when adpressed against the body, it never reaches the anal fin in larger individuals. Anal fin with iv+16(1), 17(3), 18(5*), 19(16), 20(12*), 21(5), or 22(1) rays, mature males with numerous small hooks per ray on the anal fin. Caudal fin bifurcated, with i+17+i rays and lobes of similar size. Adipose fin present.

Cycloid scales, posterior margin lacking circuli, with 4–17 radii, generally more numerous in larger individuals. Lateral line variable, being complete (21), incomplete (12**), or interrupted (15). Longitudinal series with 33(13*), 34(27*), 35(16), or 36(10) scales. Perforated scales along lateral series with 10(1), 11(2), 15(1), 17(3), 18(2), 20(1*), 21(1*), 24(3), 26(1), 28(3), 29(1), 30(2), 31(3), 32(2), 33(6), 34(8), 35(5), or 36(3) scales. Transverse scale rows 5(28*) or 6(44*) above and 4(53**) or 5(18) below the lateral line. Predorsal series with 10(2), 11(12), 12(13**), or 13(6) scales. Circumpeduncular series with 12(5), 13(12), or 14(15**) scales. Anal-fin base series with 4(15), 5(5), 6(6), or 7(1) scales.

**Color in alcohol.** Dorsal region of body, head, and tip of snout dark brown. Lateral region of body yellowish-brown or slightly silvery in its anterior portion. Ventral region of body yellowish-brown, sometimes with orange to reddish reflections. Infraorbital and opercular regions are silvery, with a dark chromatophore blotch on the opercle, more discreet in some individuals. Reticulated pattern of chromatophores (restricted to posterior margin of scales) on the anterior region of body below the lateral line. Scattered chromatophores between the lateral line and the base of the anal fin. Conspicuous humeral blotch extending 2–3 scales above and 1–2 scales below the lateral line, with upper margin wider than the lower. Usually, a clear area occurs after the humeral blotch with a reticulated pattern of chromatophores, followed by a second diffuse humeral blotch. In a few individuals, this blotch is absent, possibly due to the preservation process. Lateral stripe extending from the second humeral blotch to the median caudal-fin rays, silvery in its anterior portion and becoming darker toward the caudal peduncle, where it elongates dorsoventrally forming a distinct blotch on the caudal peduncle margin. Rayed fins hyaline, usually with few chromatophores along fin-ray margins. Adipose fin hyaline with a scattered chromatophore pattern.

**Color in life.** Dorsal region of body and head dark, brownish green on dorsum and becoming olive-green toward the flanks. Area around lateral line silvery, turning whitish to yellowish on ventral region. Pectoral fins vary from slightly yellowish to orange. Pelvic fins range from yellowish to intensely reddish. Dorsal fin yellowish green, becoming reddish toward its distal portion. Adipose fin yellowish to orange. Anal fin reddish near its base, fading distally. Caudal fin with broad reddish and yellowish margins surrounding the dark lateral stripe.

**Distribution.** *Psalidodon santae* comb. nov. is found east of the São Francisco River, in the upper São Francisco, Jequitinhonha, and Doce river basins, and possibly in the middle São Francisco. In the eastern portion of the Upper São Francisco River basin, we observed *P. santae* to be sympatric with *P. rivularis* in at least two localities: the headwaters of the das Velhas river in Ouro Preto, Minas Gerais, and in different tributaries of the Cipó River within Serra do Cipó National Park (Figure 1).

**Sexual dimorphism.** Like *P. rivularis*, reproductive males of *P. santae* have bone hooks on the anal, pectoral, and pelvic fins, from the last unbranched ray to the 15th branched ray of the anal fin, from the last unbranched ray to the 8th branched ray of the pectoral fin, and from the 3rd to the 5th branched ray of the pelvic fin (see Oliveira [14]).

**Remarks.** Here, we define *Psalidodon santae* comb. nov. as only the M2 individuals of *P. rivularis* sensu lato found east of the São Francisco River, (Figure 4, green clade, Velhas and Cipó specimens, both belonging to the drainage of the das Velhas River). *Hemigrammus santae* Eigenmann 1907 was described by Eigenmann based on a lot containing four syntypes of *Tetragonopterus rivularis* Lütken 1875 (USNM 44960). The specimens comprised two varieties of “*T.*” *rivularis*, with two specimens each, distinguished by the presence of either a complete or an interrupted lateral line [6]. From these, Eigenmann described the new species based on the variety with an interrupted lateral line (*Tetragonopterus rivularis* var. interrupta Lütken 1875), assigning the specimens to catalog number USNM 55652.

Years later, Eigenmann himself transferred the species to the genus *Hyphessobrycon*, using the naked caudal fin in *Hyphessobrycon* as a diagnostic character between the two genera [56]. However, Eigenmann did not consider an important aspect when proposing the species: Lütken [5] had noted in his work that he did not consider the interruption of the lateral line a valid character for naming a new species, since in many cases this feature was inconsistent between the two sides of the same specimen of “*T.*” *rivularis*. This misunderstanding was perpetuated in subsequent works, such as the fish identification manual for the Três Marias region [69], where in the identification key for fishes of the São Francisco River basin, an incomplete lateral line leads to the genera *Hemigrammus* and *Hyphessobrycon*, and a complete lateral line to the genus *Astyanax*, which at that time included *P. rivularis*.

Although we consider *P. santae* (Eigenmann 1907) to be a valid species, we emphasize that it is not the lateral line that distinguishes it from *P. rivularis* (Lütken 1875), since even in the type series there are specimens belonging to *P. rivularis* sensu stricto that have an interrupted lateral line. It is worth noting that in recent years, several *Hyphessobrycon* species have been transferred to the genus *Psalidodon*, increasing the number of species in the group with specimens exhibiting an interrupted lateral line [2,11].

The analysis of the types of *Astyanax turmalinensis* Triques, Vono and Caiafa 2003, shows that this taxon corresponds to the same species as *P. santae* (Eigenmann 1907). Because they were assigned to different genera, Triques et al. [7] and Triques and Queiroz [8] did not provide diagnoses differentiating these species. Therefore, we consider that the overlap in meristic characters, morphometrics, and spatial distribution justifies the synonymy of both taxa. However, since we did not analyze any *A. turmalinensis* specimens from the Doce River, and we did not obtain genetic data for specimens from the Jequitinhonha River, we suggest that further studies be conducted with *A. turmalinensis* species from these locations, as this is crucial for the validity of this species.

Regarding the morphological diagnosis of *P. santae* (Eigenmann 1907), and *P. rivularis* (Lütken 1875), the main distinguishing features are the lower number of scales along the lateral series in *P. santae* (33–36 vs. 37–39) and the higher chromosome count (2*n* = 50 chromosomes vs. 2*n* = 46 chromosomes). Other differences between these species include body coloration and morphometric traits, as noted by Eigenmann [6], with *P. santae* exhibiting a darker body, more colorful fins, and generally a taller body, with body height ranging from 31.15% to 38.48% (or possibly more) of standard length.

Additional characters noted by Triques and Queiroz [8] in the diagnosis of *A. turmalinensis* and “*Astyanax*” *rivularis* may also be used, though with caution: the origin of the pectoral fin is generally more anterior in *P. rivularis*, situated near the vertical at the midpoint of the opercle in this species, whereas in *P. santae* it originates posterior to the opercle; the region of greatest body height is generally positioned near the vertical at the origin of the pelvic fin in *P. santae* and near the vertical at the middle of the pectoral fin in *P. rivularis*, although this trait may reverse in relatively taller specimens of *P. rivularis* (body height > 30.50% of standard length) and relatively shorter specimens of *P. santae* (body height < 33.00% of standard length); the chromatophore pattern after the first humeral spot is generally reticulated (chromatophores restricted to the posterior edge of the scales) in *P. santae* and dispersed in *P. rivularis*.

Triques and Queiroz [8] also noted the presence of two humeral spots in *P. santae* versus one in *P. rivularis*. Here, we emphasize that, in fact, we observe specimens with two spots in both species, and the absence of the second spot often results from it being less distinct and lost during preservation, although it should be noted that in live or freshly fixed specimens the second spot tends to be more conspicuous in *P. santae*.

**Material Examined.** 74 specimens (four from photographs), all from the state of Minas Gerais, Brazil. **Types: São Francisco River Basin. Velhas River sub-basin:** ZMUC P241372 (photograph), paralectotype of *Tetragonopterus rivularis*, 1 specimen, standard length 70.1 mm, municipality of Lagoa Santa–MG, J. T. Reinhardt, 1847–1870. ZMUC P241376 (photograph), paralectotype of *Tetragonopterus rivularis*, 1 specimen, standard length 35.7 mm, municipality of Lagoa Santa–MG, J. T. Reinhardt, 1847–1870. USNM 55652 (photograph), syntypes of *Hemigrammus santae*, 2 specimens, standard length 32.5–54.1 mm, municipality of Lagoa Santa–MG, J. T. Reinhardt, 1847–1870. **Jequitinhonha River Basin:** DZUFMG 005, holotype of *Astyanax turmalinensis*, 1 specimen, standard length 48.2 mm, municipality of Turmalina–MG, village of Peixe-Crú, Divisão stream, right tributary of the Jequitinhonha River, 17°07′ S 42°57′ W, V. Vono, May 1989. DZUFMG 009, paratypes of *Astyanax turmalinensis*, 19 of 25 specimens, standard length 33.5–54.9 mm, same locality as holotype, V. Vono, August 1989. **Non-types. São Francisco River Basin. Velhas River sub-basin:** LaGEEvo-32, 21 specimens, standard length 31–49 mm, municipality of Ouro Preto–MG, Velhas River, 20°20′38.4″ S 43°29′58.4″ W, I.H.R. Oliveira, I. B. da Silva, P. M. de Assis, L. G. P. Pimentel, R. Pasa, 15 September 2023. **Cipó River sub-basin:** LaGEEvo-45, 9 specimens, standard length 43–53 mm, Serra do Cipó National Park–MG, Bandeirinhas Canyon, 19°25′8.33″ S 43°34′12.37″ W, M. L. C. B. de Campos, R. R. Rocha, S. V. Resende, F. Sassi, October 2017. LaGEEvo-47, 6 specimens, standard length 42–49 mm, Serra do Cipó National Park–MG, Bandeirinhas stream, 19°24′32.65″ S 43°34′35.31″ W, M. L. C. B. de Campos, R. R. Rocha, S. V. Resende, F. Sassi, October 2017. LaGEEvo-49, 8 specimens, standard length 41–59.5 mm, meeting of Mascates and Bocaina rivers, 19°20′49.68″ S 43°36′20.42″ W, I. H. R. Oliveira, I. B. da Silva, P. M. de Assis, L. G. P. Pimentel, R. A. S. Soares, G. F. da Fonseca, G. F. Matos, V. G. de Miranda, B. Alonso, 9 October 2023. LaGEEvo-58, 4 specimens, standard length 56–72 mm, Bandeirinhas Canyon, 19°25′8.33″ S 43°34′12.37″ W, I. H. R. Oliveira, I. B. da Silva, P. M. de Assis, L. G. P. Pimentel, R. A. S. Soares, G. F. da Fonseca, G. F. Matos, V. G. de Miranda, B. Alonso, 11 October 2023. LaGEEvo-62, 1 specimen, standard length 63 mm, Serra do Cipó National Park, Farofa waterfall trail, 19°23′6.52″ S 43°35′12.28″ W, I. H. R. Oliveira, I. B. da Silva, P. M. de Assis, L. G. P. Pimentel, R. A. S. Soares, G. F. da Fonseca, G. F. Matos, V. G. de Miranda, B. Alonso, 10 October 2023.

#### 3.4.3. **New Species: *Psalidodon terezinhae* sp. nov.**

**Figure 8:**
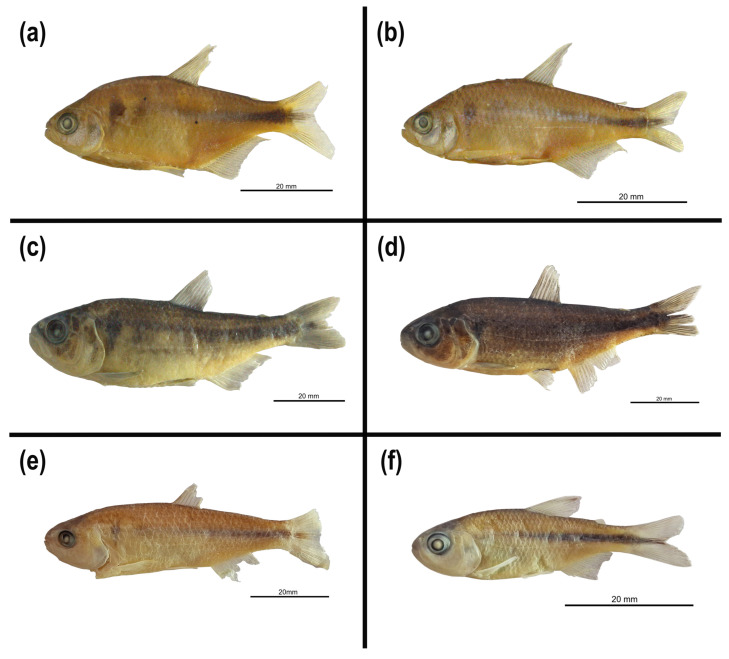
*P. terezinhae* (**a**) holotype, LaGEEvo 27-4521, 53.0 mm SL, Lage stream, Abaeté River drainage and (**b**) LaGEEvo 35-529, 45.0 mm SL, Funchal River, Indaiá River drainage. *P. velhochico* (**c**) holotype, LaGEEvo 28-5079, 77.0 mm SL, Rasga Canga Waterfall, São Francisco River drainage and (**d**) LaGEEvo 51-4999, 75.0 mm SL, Casca d’Anta Waterfall, São Francisco River drainage. *P. paiva* (**e**) holotype, LaGEEvo 29-2072, 62.0 mm SL, Bonito stream, Borrachudo River drainage and (**f**) LaGEEvo 55, 34.5 mm SL, Usina do Abaeté dam Lagoon, Abaeté River drainage.

(Figure 8a,b; Table 8)

urn:lsid:zoobank.org:act:83020156-625D-44B4-8197-BDCD32F61FE8

**Holotype.** LaGEEvo-27 (Voucher: LaGEEvo-4521). 53.0mm SL, female, Lage stream, Abaeté River drainage, Arapuá, Minas Gerais state, Brazil, 19°1′25.18″ S 46°6′18.74″ W, I.H.R. Oliveira, P. M. de Assis, T. da S. Ramos, 5 November 2022.

**Paratypes.** All specimens are from the state of Minas Gerais, Brazil. **São Francisco River Basin. Abaeté River sub-basin:** LaGEEvo-31, 22 specimens, standard length 38–54 mm, municipality of Arapuá–MG, Lage stream, 19°1′25.18″ S 46°6′18.74″ W, I.H.R. Oliveira, P. M. de Assis, T. da S. Ramos, 5 November 2022, collected with the holotype.

**Non-type material.** All specimens are from the state of Minas Gerais, Brazil. **São Francisco River Basin. Abaeté River sub-basin:** LaGEEvo-42, 15 specimens, standard length 36.5–49 mm, municipality of Arapuá–MG, Lage stream, 19°1′25.18″ S 46°6′18.74″ W, P. Penteado, D. Reis, Denis, Paloma, Wanessa, 23 July 2010. **Indaiá River sub-basin:** LaGEEvo-35, 6 specimens, standard length 35–54 mm, municipality of São Gotardo–MG, Funchal River, 19°24′9.54″ S 46°0′4.61″ W, I.H.R. Oliveira, I. B. da Silva, P. M. de Assis, L. G. P. Pimentel, 2 December 2023. LaGEEvo-38, 1 specimen, standard length 59 mm, municipality of São Gotardo–MG, Funchal River, 19°24′9.54″ S 46°0′4.61″ W, I.H.R. Oliveira, I. B. da Silva, L. Fainé, J. Godoy, 4 May 2019.

**Diagnosis.** *Psalidodon terezinhae* sp. nov. differs from most of its congeners by the following combination of characters: 18–22 branched anal-fin rays (vs. 23 or more, or 17 or fewer), 33–36 scales in the lateral series (vs. 37 or more), and body depth 33.9–38.9% of standard length (vs. less than 33.9% or greater than 38.9%). Among the remaining species, *P. terezinhae* differs from *P. anisitsi*, *P. dissimilis, P. hermosus*, *P. ita*, *P. jequitinhonhae*, *P. laticeps*, *P. minor*, *P. scabripinnis*, *P. serratus*, and *P. vermilion* by having two humeral spots (vs. one); from *P. bifasciatus*, *P. laticeps*, *P. scabripinnis*, *P. serratus*, *P. togoi*, and *P. troya* by possessing a vertically elongate humeral spot (vs. a horizontally elongate humeral spot with an anterior vertical extension); from *P. eigenmanniorum*, *P. endy*, and *P. pampa* by the pectoral-fin tip not reaching the pelvic-fin origin (vs. reaching) and the pelvic-fin tip not reaching the anal-fin origin (vs. reaching); and from *P. goyanensis*, *P. ojiara*, *P. powelli*, and *P. rioparanaibanus* by the presence of a reticulated chromatophore pattern on the anterior portion of the body below the lateral line (vs. a diffuse pattern). *Psalidodon terezinhae* further differs from *P. dissensus*, *P. puka*, and *P. pynandi* by having tricuspid outer premaxillary teeth (vs. pentacuspid in *P. dissensus* and tetra- to heptacuspid in *P. pynandi*), and maxillary teeth with 3–5 cusps (vs. 7 in *P. dissensus*, 7–9 in *P. puka*, and 5–7 in *P. pynandi*); from *P. biotae* by having 4–8 scales along the base of the anal fin (vs. 9–18); from *P. chico* by having pectoral-fin length 17.7–20.8% of standard length (vs. 22.3–25.8%) and upper jaw length 33.8–45.7% of head length (vs. 21.9–28.3%); from *P. hamatus* by having a predorsal length 47.2–53.4% of standard length (vs. 57.1–63.4%); and from *P. leonidas* by an interorbital width 30.1–39.6% of head length (vs. 26.9–30.4%). Within the *P. rivularis* species complex, *P. terezinhae* differs by having 33–36 scales in the lateral series (vs. 37–39 in *P. rivularis* and *P. velhochico*), 2*n* = 50 chromosomes (vs. 2*n* = 46 in *P. rivularis*), a reticulated chromatophore pattern on the anterior portion of the body below the lateral line (vs. a diffuse pattern in *P. velhochico*), a greater body depth (34.0–38.9% vs. 25.2–33.1% in *P. rivularis*, 28.1–33.4% in *P. velhochico*, and 28.6–32.7% in *P. paiva*), a shorter postorbital length (8.6–11.0% vs. 13.8–17.3% in *P. velhochico* and 12.2–14.5% in *P. paiva*), and a longer anal-fin base length (22.5–29.2% vs. 15.5–21.6% in *P. paiva*). Although no discrete non-overlapping character was found to distinguish *P. terezinhae* from *P. santae*, the two species can be differentiated by overlapping yet statistically distinct morphometric traits, including postorbital length (8.6–11.0%, mean 9.9% in *P. terezinhae* vs. *10.3–14.6%,* mean 11.9% in *P. santae*) and head length (21.6–26.2%, mean 23.9% in *P. terezinhae* vs. *23.1–30.1%,* mean 26.7% in *P. santae*).

**Description.** Morphometric data are available in Table 8. Body compressed, region of greatest body depth located vertically near the origin of the pelvic fin. Dorsal profile of head slightly convex from tip of upper lip to vertical through anterior nostril; slightly convex from this point to vertical through posterior margin of eye, then slightly concave from this point to supraoccipital process. Convex from supraoccipital process to base of last dorsal-fin ray; slightly convex from dorsal fin to adipose fin, and slightly concave between adipose fin and base of uppermost caudal-fin ray. Ventral profile from tip of snout to base of pelvic fin convex; from pelvic fin to base of first anal-fin ray continuously convex or straight; base of anal fin straight; and slightly concave between adipose fin and base of lowermost caudal-fin ray.

Terminal mouth, with lower jaw projecting slightly beyond upper jaw. Premaxilla with two series of teeth, the outer with 3(5), 4(16*), or 5(2) tricuspid teeth and the inner with 3(1), 4(16*), or 5(5) teeth bearing 5–7 cusps. Maxilla with 1(14*) or 2(7) small tricuspid or pentacuspid teeth. Dentary with teeth decreasing abruptly in size, 4(9*) or 5(14) large teeth with 3–5 cusps. In all teeth, the central cusps are larger than the lateral cusps. Posterior extension of maxilla reaching vertical through anterior margin of orbit.

Dorsal fin with ii+9 rays, first unbranched ray about half the length of the second. Pectoral fin with i+11(10), 12(14), or 13(5*) rays, its origin near vertical through middle of opercle; when adpressed to body, never reaching pelvic fin in larger individuals. Pelvic fin with i+6(3) or 7(24*) rays, its origin anterior to vertical through last dorsal-fin ray; when adpressed to body, never reaching anal fin in larger individuals. Anal fin with iv+18(1), 19(4), 20(8*), 21(10), or 22(3) rays. Caudal fin forked, with i+17+i rays, lobes of similar size. Adipose fin present.

Cycloid scales, circuli absent on posterior margin of scales, 4–13 radii on scales, generally more numerous in larger individuals. Lateral line variable, either complete (22*), incomplete (1) or interrupted (10). Lateral series with 33(5), 34(5), 35(12*), or 36(11) scales. Perforated scales along lateral series with 26(1), 29(2), 31(2), 32(2), 33(5), 34(3), 35(10*), or 36(8) scales. Transverse series with 5(10) or 6(23*) scales above lateral line and 4(4) or 5(29*) below. Predorsal series with 10(5), 11(13), or 12(11*) scales. Circumpeduncular series with 12(7), 13(11), or 14(10*) scales. Scales along base of anal fin 4(2), 5(6), 6(8), 7(12*), or 8(1).

**Color in alcohol.** Dorsal region of body, head, and tip of snout dark brown. Lateral region of body yellowish-brown or slightly silvery in its anterior portion. Ventral region of body yellowish-brown. Infraorbital and opercular regions silvery, with a dark blotch of chromatophores on the opercle, sometimes more discrete in some individuals. Reticulated pattern of chromatophores (restricted to posterior margin of scales) on anterior region of body below lateral line. Scattered chromatophores between lateral line and base of anal fin. Conspicuous humeral blotch extending 2–3 scales above and 1–2 scales below lateral line, with upper margin broader than lower. Clear area posterior to humeral blotch with reticulated pattern of chromatophores, followed by a second diffuse humeral blotch, which may be faint or absent due to preservation. Lateral stripe extending from second humeral blotch to median rays of caudal fin, silvery in its anterior portion and becoming darker towards caudal peduncle, where it elongates dorsoventrally forming a distinct blotch on caudal peduncle margin. Rays of fins hyaline, usually with few chromatophores along margins. Adipose fin hyaline.

**Color in life.** Dorsal region of body, head, and upper maxilla dark, brownish green on dorsum, becoming olive-green toward flanks. Lower maxilla yellowish. Infraorbital and opercular regions silvery with several dark chromatophore punctuations. First humeral blotch dark, distinct, and vertically elongated. Area around lateral line silvery with greenish reflections. Ventral region of body white with orange or yellowish reflections. Pectoral fins range from slightly yellowish to orange. Pelvic fins range from yellowish to strongly reddish. Dorsal fin yellowish green, becoming reddish toward the tip. Adipose fin yellowish or orange. Anal fin reddish on its proximal portion, fading distally. Caudal fin with broad reddish and yellowish margins surrounding the dark lateral stripe.

**Distribution.** *Psalidodon terezinhae* was collected in only two localities, the Lage stream and the Funchal River, tributaries of the Abaeté and Indaiá rivers, respectively. Thus, this species is endemic to the western portion of the Upper São Francisco River basin, being sympatric with *P. rivularis* at both sites (Figure 1).

**Etymology.** The specific epithet honors Terezinha Aparecida Rodrigues (1947–2015), the late grandmother of the first author of this work, who was also honored in the dedication of the doctoral thesis that served as the basis for this article. A genitive name.

**Remarks.** Here, we define *Psalidodon terezinhae* sp. nov. as only the M2 individuals of *P. rivularis* sensu lato found west of the São Francisco River (Figure 4, green clade, Lage and Funchal specimens). *P. terezinhae* is extremely closely related to its counterpart from the eastern portion of the Upper São Francisco River (*P. santae*), both from morphological and karyotypic perspectives, presenting 2*n* = 50 chromosomes with the same number of metacentric and subtelocentric chromosomes as *P. santae* (8 chromosomes of each morphology). Regarding the number of submetacentric and acrocentric chromosomes, the variation observed among specimens from the two sampled localities is even greater than that observed between them and *P. santae*: 24 submetacentric and 10 acrocentric chromosomes in specimens from the Lage stream, 20 submetacentric and 14 acrocentric chromosomes in the Funchal River, and 22 submetacentric and 12 acrocentric chromosomes in *P. santae* from the confluence of the Mascates and Bocaina rivers, tributaries of the Cipó River (Figure 3).

The main evidence supporting the proposal of a new species for this OTU is the lack of monophyly between these specimens and their counterparts from the eastern São Francisco River in any of the phylogenetic reconstructions performed (Figure 4), as well as morphometric differences indicating a comparatively smaller cranial region than in *P. santae*, a pattern that is reflected in our Random Forest analysis, in which most specimens were correctly classified according to postorbital length (Figure 5).

#### 3.4.4. **New Species: *Psalidodon velhochico* sp. nov.**

(Figure 8c,d; Table 8)

urn:lsid:zoobank.org:act:108F6079-0D6C-4028-91A3-6FCAD7A873EA

**Holotype.** LaGEEvo-28 (Voucher: LaGEEvo-5079). 77.0mm SL, female, upper part of Rasga Canga Waterfall, São Francisco River drainage, Serra da Canastra National Park, Minas Gerais state, Brazil, 20°10′39.13″ S 46°33′33.68″ W, I.H.R. Oliveira, I. B. da Silva, P. M. de Assis, L. G. P. Pimentel, R. A. S. Soares, T. da S. Ramos, J. Godoy, S. S. N. Pereira, 29 July 2023.

**Paratypes.** All specimens are from the Serra da Canastra National Park, state of Minas Gerais, Brazil. **São Francisco River basin:** LaGEEvo-50, 15 specimens, standard length 49–89 mm, Serra da Canastra National Park–MG, upper part of Rasga Canga Waterfall, 20°10′39.13″ S 46°33′33.68″ W, I.H.R. Oliveira, I. B. da Silva, P. M. de Assis, L. G. P. Pimentel, R. A. S. Soares, T. da S. Ramos, J. Godoy, S. S. N. Pereira, 29 July 2023, collected with the holotype. LaGEEvo-53, 23 specimens, standard length 50–93 mm, Serra da Canastra National Park–MG, lower part of Rasga Canga Waterfall, 20°10′39.13″ S 46°33′33.68″ W, I.H.R. Oliveira, I. B. da Silva, P. M. de Assis, L. G. P. Pimentel, R. A. S. Soares, T. da S. Ramos, J. Godoy, S. S. N. Pereira, 29 July 2023.

**Non-type material.** All specimens are from the Serra da Canastra National Park, state of Minas Gerais, Brazil. **São Francisco River basin:** LaGEEvo-51, 34 specimens, standard length 43–83 mm, Serra da Canastra National Park–MG, Casca d’Anta Waterfall, 20°18′2.24″ S 46°31′18.99″ W, I.H.R. Oliveira, I. B. da Silva, P. M. de Assis, L. G. P. Pimentel, R. A. S. Soares, T. da S. Ramos, J. Godoy, S. S. N. Pereira, 28 July 2023. LaGEEvo-52, 34 specimens, standard length 40–81 mm, Serra da Canastra National Park–MG, historical source of the São Francisco River, 20°14′4.79″ S 46°26′29.38″ W, I.H.R. Oliveira, I. B. da Silva, P. M. de Assis, L. G. P. Pimentel, R. A. S. Soares, T. da S. Ramos, J. Godoy, S. S. N. Pereira, 28 July 2023.

**Diagnosis.** *Psalidodon velhochico* sp. nov. differs from most of its congeners by the following combination of characters: 15–20 branched anal-fin rays (vs. 21 or more, or 14 or fewer), 37–39 scales in the lateral series (vs. 36 or fewer, or 40 or more), and body depth equal to or less than 33.4% of standard length (vs. 33.5% or greater). Among the remaining species, *P. velhochico* differs from *P. dissimilis*, *P. hermosus*, *P. laticeps*, *P. paranae*, *P. scabripinnis*, *P. serratus*, and *P. varzeae* by having two humeral spots (vs. one); from *P. laticeps*, *P. leonidas*, *P. scabripinnis*, *P. serratus*, *P. troya*, and the remaining species of the *P. rivularis* complex by the presence of a diffuse chromatophore pattern on the anterior region of the body below the lateral line (vs. a reticulated pattern); from *P. eigenmanniorum* and *P. xiru* by having a shorter anal-fin base length (16.2–23.3% of SL vs. 23.7–35.6% in *P. eigenmanniorum* and 23.3–27.4% in *P. xiru*); and from *P. brachypterygium* and *P. cremnobates* by having a shorter dorsal-fin length (15.8–21.4% of SL vs. 23.6–29.3% in *P. brachypterygium* and 22.4–28.4% in *P. cremnobates*). *P. velhochico* further differs from *P. rivularis* sensu stricto by having 2*n* = 50 chromosomes (vs. 2*n* = 46) and a longer postorbital length (13.8–17.3% of SL vs. 9.4–13.1%); and from *P. santae*, *P. terezinhae* sp. nov., and *P. paiva* sp. nov. by having 37–39 scales in the lateral series (vs. 33–36).

**Description.** Morphometric data are available in Table 8. Body compressed, greatest body depth generally located vertically between the origin and the end of the pectoral fin. Dorsal profile of the head slightly convex between the tip of the upper lip and the vertical anterior to the nostril; continuously convex from this point to the supraoccipital process. Profile convex from the supraoccipital process to the base of the last dorsal-fin ray; slightly convex from the dorsal to the adipose fin, and slightly concave between the adipose fin and the base of the upper caudal-fin ray. Ventral profile from the tip of the snout to the vertical through the end of the maxilla slightly convex, and from this point to the base of the first anal-fin ray markedly convex; anal-fin base straight; and slightly concave between the adipose fin and the base of the lower caudal-fin ray.

Terminal mouth, with lower jaw slightly projecting beyond the upper jaw. Premaxilla with two series of teeth, the outer with 3(3), 4(25), 5(10*), or 6(1) tricuspid teeth and the inner with 4(17) or 5(22*) teeth bearing 5–7 cusps. Maxilla with 1(20*) or 2(15) small tricuspid or pentacuspid teeth. Dentary with teeth decreasing abruptly in size, 4(8), 5(25*) or 6(4) large teeth with 4–6 cusps. In all teeth, the central cusps are larger than the lateral cusps. Maxilla extending posteriorly beyond the vertical through the anterior margin of the orbit.

Dorsal fin with ii+9 rays, the first unbranched ray about half the length of the second. Pectoral fin with i+12(16), 13(16*) or 14(5) rays, its origin close to the vertical through the middle of the opercle, when adpressed to the body it never reaches the pelvic fin. Pelvic fin with i+7 rays, its origin anterior to the vertical through the last dorsal fin ray, when adpressed to the body it never reaches the anal fin. Anal fin with iv+15(1), 16(18), 17(44*), 18(20), 19(4), or 20(1) rays. Caudal fin forked with i+17+i rays and lobes of similar size. Adipose fin present.

Cycloid scales, circuli absent on posterior margin, with 7–18 radii, usually more numerous in larger individuals. Lateral line variable, complete (70*), incomplete (5), or interrupted (31). Lateral series with 37(43), 38(29), or 39(24*) scales. Perforated scales along lateral series with 26(1), 29(1), 30(2), 31(2), 34(4), 35(3), 36(16), 37(29), 38(34), or 39(14*) scales. Transverse series with 5(60) or 6(47*) scales above the lateral line and 4(9) or 5(97*) below. Predorsal series with 11(4), 12(17), or 13(18*) scales. Scales around caudal peduncle 12(11), 13(15), or 14(11*). Scales at the base of the anal fin 3(9), 4(10), 5(10*), 6(5), or 7(2).

**Color in alcohol.** Dorsal region of the body, head, and tip of the snout dark brown. Lateral region of the body is yellowish-brown or dark brown due to the large number of chromatophores scattered across the scales. Ventral region of the body yellowish-brown. Infraorbital and opercular regions yellowish-brown, with dark chromatophore spots on the opercle and infraorbitals around the orbit, sometimes more discreet in some individuals. Dispersed chromatophore pattern (not restricted to the posterior margin of the scales) in the anterior region of the body below the lateral line. Dispersed chromatophore pattern between the lateral line and the base of the anal fin. Conspicuous humeral spot extending 3–4 scales above the lateral line and 1–2 scales below, with the upper margin slightly wider than the lower margin. In some individuals, a second diffuse humeral spot can be observed, often difficult to distinguish due to its fusion with the lateral stripe and the darker body coloration in this species. Lateral stripe extending from the second humeral spot to the median rays of the caudal fin, becoming progressively thicker toward the caudal peduncle, where it expands dorsoventrally forming a distinct blotch at the margin of the caudal peduncle. Rayed fins hyaline, generally with few chromatophores along the margin of the rays. Adipose fin hyaline with a dispersed chromatophore pattern.

**Color in life.** Dorsal and lateral regions of the body, head, and upper maxilla dark, with a brownish-green hue extending from the dorsum to the region below the lateral line. Lower maxilla yellowish. First humeral spot dark, distinct, and vertically elongated. Area surrounding the lateral stripe with golden reflections posteriorly and greenish reflections anteriorly. Ventral region of the head and infraorbitals ranging from whitish to yellowish tones. Ventral region of the body white with yellowish reflections. Pectoral, pelvic, and anal fins intensely reddish. Dorsal and adipose fins range from yellowish to intensely reddish. Caudal fin with broad reddish and yellowish margins surrounding the dark lateral stripe.

**Distribution.** *Psalidodon velhochico* was collected in only three localities, all tributaries of the headwaters of the São Francisco River within Serra da Canastra National Park: the historical headwater of the São Francisco River, Rasga Canga Waterfall (upper and lower sections), and Casca d’Anta Waterfall (upper section) (Figure 1).

**Etymology.** The specific epithet *velhochico* is used as a noun in apposition and refers to the popular nickname of the São Francisco River in Brazil, affectionately called “Velho Chico” (“Old Chico”) by Brazilians.

**Remarks.** Here, we define *Psalidodon velhochico* sp. nov. as only the M3 individuals of *P. rivularis* sensu lato (Figure 4, yellow clade). *P. velhochico* sp. nov. is clearly a distinct species from the other OTUs within *P. rivularis* sensu lato, supported by all sources of data used in this study, and it is also the only one not showing sympatry with any of the others. In addition to the previously mentioned chromatophore pattern on the anterior body region, this OTU has 2*n* = 50 chromosomes (vs. 2*n* = 46 in *P. rivularis*), with a markedly distinct karyotypic formula: 10 metacentric chromosomes (vs. 8 in the other OTUs), 18 submetacentric chromosomes (vs. 20 or more), 6 subtelocentric chromosomes (vs. 8–10), and 16 acrocentric chromosomes (vs. 14 or fewer). Some specimens also bear a distinctive large metacentric supernumerary chromosome, bigger than any other chromosome in the karyotype, which has otherwise been observed only in *P. paiva* sp. nov.

From a molecular perspective, this OTU is phylogenetically close to *P. rioparanaibanus* and *P.* aff*. paranae*, forming a well-supported clade herein referred to as the “Paranaíba–Canastra” clade. This relationship is also reflected in morphological similarities, since both species exhibit a dispersed chromatophore pattern on the anterior body region below the lateral line. *P. velhochico* can be distinguished from *P. rioparanaibanus* by having 37–39 scales along the longitudinal series (vs. 34–36), and by lacking the dorsal pattern of alternating silvery and dark scales that characterizes *P. rioparanaibanus*. Furthermore, as in the other species within *P. rivularis* sensu lato, the lateral line in *P. velhochico* is variable, a trait not observed in the remaining species of the “Paranaíba–Canastra” clade.

#### 3.4.5. **New Species: *Psalidodon paiva* sp. nov.**

(Figure 8e,f; Table 8)

urn:lsid:zoobank.org:act:C538848A-E345-4F81-BC46-FA96CED0F723

**Holotype.** LaGEEvo-29 (Voucher: LaGEEvo-2072). 62.0mm SL, male, Bonito Stream, Borrachudo River drainage, municipality of Tiros, Minas Gerais state, Brazil, 18°48′44.7″ S 45°45′52.2″ W, P. Penteado, Denis, Gabriel, Rafael, 20 July 2010.

**Paratypes.** All specimens are from the state of Minas Gerais, Brazil. **São Francisco River Basin. Borrachudo River Sub-basin:** LaGEEvo-39, 5 specimens, standard length: 52 mm–77 mm, municipality of Tiros–MG, Bonito Stream, 18°48′44.7″ S 45°45′52.2″ W, P. Penteado, Denis, Gabriel, Rafael, 20 July 2010, collected with the holotype. **Abaeté River Sub-basin:** LaGGEvo-55, 10 specimens, standard length: 31.5 mm–44 mm, municipality of Rio Paranaíba–MG, lagoon of the Abaeté Dam, 19°12′35.69″ S 46°6′33.79″ W, I. H. R. Oliveira, I. B. da Silva, M. A. da Silva, 7 July 2017.

**Non-type material.** All specimens are from the state of Minas Gerais, Brazil. **São Francisco River Basin. Abaeté River Sub-basin:** LaGGEvo-54, 10 specimens, standard length: 42 mm–72 mm, municipality of Rio Paranaíba–MG, lagoon of the Abaeté Dam, 19°12′35.69″ S 46°6′33.79″ W, W. Lopes-Silva, C. H. M. Fernandes, M. A. da Silva, A. C. M. Fernandes, 17 July 2012. LaGEEvo-57, 4 specimens, standard length: 57.5 mm–67.5 mm, municipality of Tiros–MG, Tiros Stream, 18°56′34.08″ S 45°56′18.20″ W, 2010–2012. **Paracatu River Sub-basin:** LaGEEvo-76, 9 specimens, standard length: 37 mm–45 mm, municipality of Presidente Olegário–MG, Crico Stream, 18°18′44.36″ S 46°5′44.46″ W, I. B. da Silva, M. L. C. B. de Campos, V. Augusto, S. V. Resende, 6 April 2019.

**Diagnosis.** *Psalidodon paiva* sp. nov. is distinguished from most of its congeners by the following combination of characters: 14–17 branched anal-fin rays (vs. 18 or more) and body depth equal to or less than 32.7% of standard length (vs. greater than 33%). Among the remaining species, *P. paiva* differs from *P. brachypterygium*, *P. cremnobates*, *P. goyanensis*, *P. paranae*, *P. rioparanaibanus* and *P. varzeae* by having a reticulated pattern of chromatophores on the anterior region of the body below the lateral line (vs. dispersed pattern); from *P. crenuchoides*, *P. kalunga*, and *P. pessalii* by the presence of an adipose fin (vs. absence); from *P. scabripinnis* and *P. serratus* by having a vertically elongated humeral blotch (vs. a horizontally elongated humeral blotch with a vertical anterior extension); from *P. leonidas* by having an anal-fin base length of 15.5–21.6% of SL (vs. 21.8–27.1% in *P. leonidas*); and from *P. dissimilis* by having a maxillary length of 33.8–43.5% of HL (vs. 24.3–29.2% in *P. dissimilis*). It is further distinguished from the remaining species of the *P. rivularis* species complex by having 33–36 scales in the lateral series (vs. 37–39 in *P. rivularis* and *P. velhochico*), 2*n* = 50 chromosomes (vs. 2*n* = 46 in *P. rivularis*), and a reticulated pattern of chromatophores below the anterior portion of the lateral line (vs. dispersed in *P. velhochico*). In addition, it differs from *P. terezinhae* by having a shallower body depth (28.6–32.7% of SL vs. 33.9–38.9%), a shorter anal-fin base (15.5–21.6% of SL vs. 22.5–29.2%), and a greater postorbital length (12.2–14.5% of SL vs. 8.6–11.0%). Although no discrete character was found to distinguish *P. paiva* sp. nov. from *P. santae*, the two species can be separated by overlapping but statistically distinct morphometric ranges, including body depth (28.6–32.7%, mean 30.9% in *P. paiva* vs. *31.2–38.6%,* mean 34.8% in *P. santae*) and anal-fin base length (15.5–21.6%, mean 19.3% in *P. paiva* vs. *20.6–28.5%,* mean 24.9% in *P. santae*). In addition, *P. paiva* sp. nov. has the greatest body depth at a vertical through the mid to distal portion of the pectoral fin, whereas in *P. santae* and *P. terezinhae* the greatest body depth is usually at a vertical through the origin of the pelvic fin.

**Description.** Morphometric data are available in Table 8. Body compressed, greatest body depth usually located vertically between the middle and the tip of the pectoral fin. Dorsal profile of head slightly convex between the tip of the upper lip and the vertical through the anterior nostril; slightly convex, straight, or slightly concave from this point to the supraoccipital process. Dorsal profile from the supraoccipital process to the base of the last dorsal-fin ray convex; slightly convex from the dorsal to the adipose fin, and slightly concave between the adipose fin and the base of the upper caudal-fin ray. Ventral profile from tip of snout to base of pelvic fin convex; from pelvic fin to base of first anal-fin ray continuously convex or straight; base of anal fin straight; and slightly concave between the adipose fin and the base of the lower caudal-fin ray.

Mouth terminal, lower jaw slightly projecting beyond upper jaw. Premaxilla with two series of teeth, the outer with 3(2) or 4(14*) tricuspid teeth and the inner with 4(7*), 5(8), or 6(1) teeth bearing five cusps. Maxilla with 1(12*) or 2(1) small tricuspid or pentacuspid teeth. Dentary with teeth decreasing abruptly in size, 4(7*), 5(7) or 6(2) large teeth with 3–5 cusps. In all teeth, the central cusps are larger than the lateral cusps. Posterior end of maxilla extending beyond vertical through anterior orbital margin.

Dorsal fin with ii+9 rays, first unbranched ray half the length of the second. Pectoral fin with i+12(11), 13(15), or 14(3*) rays, origin close to vertical through posterior end of opercle, never reaching pelvic fin in larger individuals when adpressed. Pelvic fin with i+7 rays, origin anterior to vertical through last dorsal-fin ray, when adpressed to body, never reaching anal fin in larger individuals. Anal fin with iv+14(5*), 15(9), 16(15), or 17(2) rays. Caudal fin forked with i+17+i rays, lobes of similar size. Adipose fin present.

Scales cycloid, posterior margin without circuli, 4–19 radii on scales, generally more numerous in larger individuals. Lateral line variable, complete (21*), incomplete (1), or interrupted (12); 33(2), 34(10), 35(14), or 36(10*) scales in lateral series. Perforated scales along lateral series with 24(1), 28(1), 29(1), 31(2), 32(1), 33(2), 34(11), 35(9), or 36(7*) scales. Transverse scale series with 5(22*) or 6(16) scales above lateral line and 4(16*) or 5(22) below. Predorsal scale series with 12(4), 13(18*), or 14(8) scales. Circumpeduncular scale series with 12(5), 13(8), or 14(17*) scales. Anal-fin base scale series with 3(14), 4(5*), 5(5), 6(2), 7(1), or 8(2) scales.

**Color in alcohol.** Dorsal region of body, head, and snout tip dark brown. Lateral region of body yellowish-brown or slightly silvery anteriorly. Ventral region of body yellowish-brown. Infraorbital and opercular regions silvery, with a faint dark blotch of chromatophores on opercle, sometimes absent in some individuals. Reticulated pattern of chromatophores (restricted to posterior margin of scales) present on anterior portion of body below lateral line. Dispersed chromatophore pattern between lateral line and anal-fin base. Conspicuous humeral blotch extending 2–3 scales above and 1–2 scales below lateral line, upper margin broader than lower. Clear area posterior to humeral blotch with few chromatophores in a dispersed pattern. Lateral stripe that begins slightly after the humeral spot and extends to the median caudal-fin rays, silvery anteriorly and becoming darker toward caudal peduncle, where it expands dorsoventrally to form a distinct blotch on caudal-peduncle margin. The most anterior portion of the dark lateral stripe resembles a second humeral spot but lacks a dorsoventral extension. Rayed fins hyaline, usually with few chromatophores along fin-ray margins. Adipose fin hyaline with scattered chromatophores.

**Distribution.** *Psalidodon paiva* sp. nov. was collected in tributaries of the Abaeté, Borrachudo, and Paracatu rivers in the western portion of the São Francisco River basin, thus occurring in the Upper and Middle São Francisco drainages. In three of the four localities where it was collected (Bonito stream, Tiros stream, and Crico stream), this species is sympatric with *P. rivularis* sensu stricto, whereas in the fourth locality (Abaeté Power Plant Dam Lagoon) it is sympatric with *A. lacustris* (Figure 1).

**Etymology.** The specific epithet is used as a noun in apposition and honors the Paiva family, whose life, portrayed in the Brazilian Oscar-winning film “Ainda Estou Aqui” (2024), was profoundly affected by the imprisonment, disappearance, and execution of the family patriarch, Rubens Paiva, during the Brazilian military dictatorship.

**Remarks.** Here, we define *Psalidodon paiva* sp. nov. as only the M4 individuals of *P. rivularis* sensu lato (Figure 4, light blue clade). *Psalidodon paiva* sp. nov. resembles *P. santae* and *P. terezinhae* sp. nov. in meristic and morphological traits; however, its morphometric proportions are more like those of *P. rivularis* and *P. velhochico* sp. nov., particularly in having a lower body depth, as observed in PCA and Random Forest analyses (Figure 3). From a cytogenetic perspective, this species has 2*n* = 50 chromosomes, differing from *P. rivularis* sensu stricto (2*n* = 46). This OTU can also be distinguished from *P. rivularis, P. santae,* and *P. terezinhae* by the higher number of subtelocentric chromosomes (10 vs. 8) (Figure 3). From a molecular perspective, excluding the mitochondrial genome dataset, this species appears to be more closely related to the “*paranae–scabripinnis*” group than to other OTUs of the *P. rivularis* sensu lato group (Figure 4).

Although we did not collect this species during the present study, it is well represented in the Ichthyological Collection of LaGEEvo UFV CRP, usually identified as “*P. rivularis* 2*n* = 50.” Prior to this study, this species already had a mitochondrial genome available in the NCBI nt database (MT428070.1), identified as the cytotype with 2*n* = 50 chromosomes of *P. rivularis* (LAGEEVO–2614) by Pasa et al. [21].

**Comparative material examined.** *Astyanax lacustris:* ZMUC P241329 (photograph), syntype of *Tetragonopterus lacustris*, 1 specimen, standard length: 51.12 mm, Lagoa Santa municipality–MG, 19°37′52″ S 43°54′07″ W, J. T. Reinhardt, 1850–1856. LaGEEvo-65, 2 specimens, standard length: 69–78.5 mm, municipality of Lagoa da Prata-MG, Retiro de Baixo stream, 20°0′15.75″ S 45°30′42.13″ W, I.H.R. Oliveira, I. B. da Silva, R. A. S. Soares, L. da C. de Santos, 6 May 2023. LaGEEvo-66, 3 specimens, standard length: 69–79 mm, municipality of Augusto de Lima-MG, Teixeira stream, 17°58′58.53″ S 44°4′26.566″ W, V. G. de Miranda, Jul 2024. LaGEEvo-71, 12 specimens, standard length: 49–59 mm, municipality of Rio Paranaíba-MG, Abaeté Power Plant Dam lagoon, 19°12′35.69″ S 46°6′33.79″ W, I.H.R. Oliveira, P. M. de Assis, L. G. P. Pimentel, R. A. S. Soares, 10 January 2022.

*Psalidodon fasciatus:* ZMUC P241291 (photograph), syntype of *Tetragonopterus cuvieri*, 1 specimen, standard length: 86.5 mm, Lagoa Santa municipality-MG, 19°37′52″ S 43°54′07″ W, J. T. Reinhardt, 1850–1856. LaGEEvo-67, 1 specimen, standard length: 98 mm, municipality of Lagoa da Prata-MG, Retiro de Baixo stream, 20°0′15.75″ S 45°30′42.13″ W, I.H.R. Oliveira, I. B. da Silva, R. A. S. Soares, L. da C. de Santos, 6 May 2023. LaGEEvo-68, 3 specimens, standard length: 68–83 mm, municipality of Augusto de Lima-MG, Teixeira stream, 17°58′58.53″ S 44°4′26.566″ W, V. G. de Miranda, Jul 2024. LaGEEvo-69, 7 specimens, standard length: 38.5–89 mm, Lagoa da Prata-MG, Santana river, 20°0′15.75″ S 45°30′42.13″ W, I.H.R. Oliveira, I. B. da Silva, R. A. S. Soares, L. da C. de Santos, 6 May 2023. LaGEEvo-70, 1 specimen, standard length: 64 mm, Três Marias-MG, Vereda Grande river, 18°19′18.62″ S 45°6′32.80″ W, R. de M. Alves, R. R. Rocha, M. A. da Silva, S. V. Resende, 18 April 2017.

*Psalidodon paranae*: LaGEEvo-72, 7 specimens, standard length: 53.5–77.5 mm, Rio Paranaíba-MG, Lava-Pés stream, 19°11′41.22″ S 46°15′7.32″ W, M. L. C. B. de Campos, F. Sassi, T. Lunardi, R. Pereira, M. Trevisanuto, November 2015. LaGEEvo-73, 3 specimens, standard length: 77–83 mm, Rio Paranaíba-MG, Lava-Pés stream, 19°11′41.22″ S 46°15′7.32″ W, M. L. C. B. de Campos, F. Sassi, T. Lunardi, R. Pereira, M. Trevisanuto, November 2015.

*Psalidodon rioparanaibanus*: LaGEEvo-12, holotype of *Psalidodon rioparanaibanus*, 1 specimen, standard length: 84 mm, Rio Paranaíba-MG, Rita stream, 19°11′15.77″ S 46°14′10.24″ W, M. A. da Silva & I. B. da Silva, 21 August 2017. LaGEEvo-13, paratypes of *Psalidodon rioparanaibanus*, 8 of 15 specimens, standard length: 43–84 mm, Rio Paranaíba-MG, Rita stream, 19°11′15.77″ S 46°14′10.24″ W, I.H.R. Oliveira, I. B. da Silva, R. L. Oliveira, G. Leles, T. Castaño, 23 September 2018. LaGEEvo-14, paratypes of *Psalidodon rioparanaibanus*, 4 specimens, standard length: 40–84 mm, Rio Paranaíba-MG, Rita stream, 19°11′15.77″ S 46°14′10.24″ W, M. A. da Silva & I. B. da Silva, 21 August 2017.

## 4. Discussion

In this study, we were able, through an integrated approach, to delimit five different OTUs within the *P. rivularis* sensu lato group distributed across several tributaries in the mesoregion of the Upper São Francisco River. Although initially surprising, this number of species is plausible when we consider that, even accounting for *P. santae*, the Upper São Francisco River harbors only four valid species of *Psalidodon* (*P. rivularis*, *P. santae* comb. nov., *P. pessalii*, and *P. fasciatus*). This number is relatively smaller than in other Brazilian hydrographic mesoregions, such as the Paranaíba River (8 spp.), the Grande River (7 spp.), and the Iguaçu River (8 spp.) [70]. In this context, we not only advance the knowledge of the biodiversity of the genus *Psalidodon* in the Upper São Francisco River, but also highlight the necessity of approaches that integrate data from different sources—i.e., molecular, phenotypic, and biogeographic—in the study of species complexes [9], since, as discussed below, all these data sources presented both merits and challenges in delimiting the different OTUs of the group.

Two of the OTUs we identified correspond to the two morphotypes described by Lütken [5]: *P. rivularis* sensu stricto and *P. santae* comb. nov., the latter being a new combination for *H. santae* (Eigenmann 1907). We also provide a new diagnosis for both species, as the lateral-line completeness mentioned by Eigenmann [6] is insufficient to diagnose these two species, or any of the five OTUs identified in this study, since all exhibit a variable lateral line, which may be complete, incomplete, or interrupted. This trait is considered paedomorphic in characids, resulting from truncation of lateral-line development, and has independently appeared multiple times in Acestrorhamphidae, including in the genus *Psalidodon*, having been reported, besides *P. rivularis* and *P. santae*, in *P. balbus*, *P. brachypterygium*, *P. cremnobates*, and *P. anisitsi* [71].

Among the morphological traits that proved useful in diagnosing the different OTUs of *P. rivularis* sensu lato, and as observed in our previous work [62], body depth was the trait with the greatest variation within the group, being also the characteristic that most distinguishes specimens of M2 (*P. santae* and *P. terezinhae*) from the other morphotypes of the group (Figure 5). The differences between M2 and the other morphotypes of *P. rivularis* sensu lato resemble Cartesian transformations related to fish morphogenesis, as discussed by D’Arcy Thompson in his work On Growth and Form [72]. These transformations are now recognized as changes in ontogenetic rates, associated with heterochrony (changes in the rate and timing of development) and heterotopy (changes in the spatial pattern of development) [73].

Since body depth in fishes is strongly influenced by environmental factors, particularly water velocity [62,74,75], the changes in body depth observed in specimens of M2 relative to the others may result from alterations in ontogenetic growth rates, allowing these specimens to occupy an ecological niche different from that of M1, with which they are sympatric at several localities in the Upper São Francisco River. This explanation has previously been proposed for other phylogenetically related fish species that exhibit discrepancies in body depth [76,77].

Another morphometric trait that proved useful in diagnosing the OTUs of *P. rivularis* sensu lato was the postorbital length (POL), and it is important to contextualize the use of POL as a morphometric character. A similar measurement (OOL) was recently employed by Engelman [78] as a method to estimate the body size of the extinct placoderm fish *Dunkleosteus terreli*. According to the author, this measurement can be used to estimate the size of a wide range of fish groups, from agnathans to bony fishes, because its size is strongly constrained, encompassing both the neurocranium and the branchial chamber. In the case of the branchial chamber, it is particularly important due to its functional demand in oxygen consumption [78].

The POL differs from OOL by excluding the orbit diameter from the measurement. An important reason for analyzing the postorbital region separately from the orbit follows a functional interpretation of cranial modularity in characiforms, in which the snout, orbit, and post-orbital region may act as partially independent modules [79], although the exact modular structure can vary even among related taxa and ideally, it needs to be tested. In this context, the snout and orbit correspond to modules already captured by traditional characid morphometric measurements [49]. The fish snout, defined from the tip of the rostrum to the anterior margin of the orbit, is known to vary even among closely related species due to its direct relationship with feeding habits [76,77,78,79]. The orbit is also known to vary in closely related fish species and has even been reported as one of the distinguishing characters of “*Astyanax turmalinensis*” [7]. Finally, post-orbital elongation is recognized as an important ontogenetic process in fishes [73], and in our previous work using geometric morphometrics, we identified that the retraction of the suborbital plate relative to the opercle was the second most variable trait in *P. rivularis* [62].

In our study, we observed that the POL was statistically different between *P. santae* and *P. terezinhae*, *P. rivularis* and *P. velhochico*, and *P. paiva* and *P. terezinhae*. Interestingly, in the first two cases, these species are geographically separated. As previously noted, *P. santae* and *P. terezinhae* inhabit opposite sides of the São Francisco River, with the former occurring east of the river (headwaters of the Velhas, Cipó, Jequitinhonha, and Doce rivers) and the latter west of the river (headwaters of the Abaeté and Indaiá rivers). Like the other species in the *Psalidodon scabripinnis* complex, these species are known to inhabit only headwater environments, and thus the main channel of large rivers acts as a natural barrier, isolating populations and promoting their chromosomal and morphological evolution [3].

This pattern is consistent with the occurrence of *P. rivularis* sensu lato in streams (such as Córrego Lage in the Abaeté River and Córrego Divisão in the Jequitinhonha River), smaller rivers (such as the Funchal River, a tributary of the Indaiá River, and the Mascates and Bocaina rivers, tributaries of the Cipó River), or headwaters of large rivers (such as the source of the Velhas River in Ouro Preto, Minas Gerais). In contrast, the main channels of the Upper São Francisco hydrographic mesoregion are predominantly inhabited by *P. fasciatus* and *A. lacustris*, with few cases of sympatry observed between these latter species and *P. rivularis* sensu lato (Figure 1).

For *P. rivularis* and *P. velhochico*, the factor of geographic isolation appears to be different. While *P. rivularis* is distributed across multiple tributaries in the mesoregion of the Upper São Francisco River, *P. velhochico* is confined to tributaries near the headwaters of the São Francisco River in the highland environments of the Serra da Canastra National Park (above 1100 m in altitude). In this context, we propose a phylogeographic explanation for the occurrence of this second OTU in the São Francisco River basin. Considering that the region where *P. velhochico* is collected in Serra da Canastra National Park borders the Paranaíba and Grande river basins, and that this OTU groups with the species of the Paranaíba River, *P. rioparanaibanus*, and *P.* aff. *paranae* in all phylogenies performed, it is possible that the presence of this OTU in the São Francisco River can be explained by headwater capture vicariant processes occurring during extensive tectonic activity in the Pliocene–Pleistocene transition [80].

This hypothesis is also supported by our time-calibrated phylogeny, in which the main clades addressed in this study show an estimated Pliocene origin. The timing of diversification, typically between 2.8 and 5.6 Mya, together with the present-day distribution of these species across both coastal drainages (such as the Doce and Jequitinhonha rivers) and adjacent continental basins (such as the São Francisco and Paraná rivers), suggests that the genus *Psalidodon*, as well as several other characids, fits within Ribeiro’s biogeographic pattern B [80], which is characterized by lineages derived from cladogenetic events that occurred at least during the Tertiary.

Thus, the existence of the “Paranaíba-Canastra” clade reported here, composed of *Psalidodon* species from the *P. scabripinnis* group, adds to several other examples of vicariance between sister or cryptic species of Acestrorhamphidae associated with the uplift of the Paranaíba Arc [44,81,82]. This geological event was responsible for the isolation of the Paranaíba and Upper São Francisco river basins and began during the Cretaceous, between 117 and 119 Mya [83]. Despite its ancient origin, a phase of neotectonic reactivation took place in the region during the late Tertiary, largely conditioning the current drainage system [83], and roughly coincides with the tMRCA we estimated for the Paranaíba–Canastra clade (3.1–5.2 Mya). Therefore, it is likely that the uplift of the Paranaíba Arc, together with neotectonic events in the São Francisco River basin, led to the formation of multiple endemic zones in high-altitude environments near the boundary of these two basins, which may explain the restricted occurrence of some species, such as *P. velhochico* and *P. rioparanaibanus* [84]. Furthermore, intense geological processes are known to generate phenomena such as headwater captures, which in turn can lead to both vicariant events and dispersal or geodispersal events [85]. Headwater captures may not only explain the presence of *P. velhochico* in Serra da Canastra, but also the presence of *P. paiva*, a species related to the “*paranae-scabripinnis*” clade, in the Upper São Francisco River basin.

Another important evolutionary aspect and diagnostic criterion in the delimitation of *P. rivularis* sensu lato OTUs is, without a doubt, their chromosomal diversity (Figure 3). Knowledge of the chromosomal diversity of *P. scabripinnis* from the São Francisco River is not new, with the first documentation of populations with different diploid numbers (2*n* = 46 and 2*n* = 50) dating back to the late twentieth century [3]. Here, we demonstrate that, in addition to karyotypic differences among the different morphotypes of *P. rivularis* sensu lato, diploid number differences distinguish specimens of M1 (*P. rivularis* sensu stricto, 2*n* = 46) from the other morphotypes (2*n* = 50). It is important to emphasize that the existence of different cytotypes/karyomorphs is not, by itself, diagnostic of distinct species, as there are known species with different chromosome numbers capable of interbreeding, resulting in multiple hybrid forms, as exemplified by *P. fasciatus* [86].

However, it is well known that changes in chromosome number can lead to post-zygotic reproductive isolation, with hybrid fertility declining and potentially reaching complete infertility as parental genomes become more genetically distant [87]. This degree of reproductive isolation appears to occur at least among the cytotypes with different chromosome numbers of *P. rivularis* sensu lato, which would explain why all observed cases of sympatry occurred in pairs, always involving specimens of *P. rivularis* sensu stricto (2*n* = 46) with specimens of another OTU, which could be *P. santae*, *P. terezinhae*, or *P. paiva* (2*n* = 50). Additional evidence lies in the fact that we did not observe intermediate forms between these cytotypes in this study. Metaphases with 48 chromosomes were rare, and subsequent analyses showed that they were either artifacts of incomplete metaphases in specimens with 2*n* = 50 chromosomes, or the result of the presence of two supernumerary chromosomes in a few specimens (n = 3) of *P. rivularis* sensu stricto from Lage stream.

It is also noteworthy that the mitochondrial genome phylogeny did not recover specimens of *P. rivularis* sensu stricto, *P. santae*, or *P. paiva* as monophyletic groups. In fact, all these specimens (except for *P. santae* from the Cipó River) were recovered within a single clade that also includes *P. terezinhae* (Figure 4a). Overall, nearly all mitochondrial genomes of *P. rivularis* sensu lato exhibit low genetic distances among them (less than 2%), which aligns the different OTUs of *P. rivularis* sensu lato with the chromosomal radiation model [88].

When comparing mitochondrial phylogeny with multilocus phylogenies (Figure 4b,c), it becomes evident that the mitochondrial phylogeny can fail to accurately recover the phylogenetic relationships within the group. This phenomenon, known as mito-nuclear discordance, can result from various factors, such as incomplete lineage sorting, retention of ancestral haplotypes, selection, sex-biased dispersal, introgression, mitochondrial capture, and hybridization [89,90,91]. For instance, a history of hybridization could explain the lack of structure among the OTUs of the group, whereas introgression with mitochondrial capture could account for the divergence observed in the mitochondrial genome of *P. santae* from the Cipó River [90]. Although indicative of important biological events in the evolution of the group, the mito-nuclear discordance observed here underscores that species delimitation and identification using mitochondrial genes—so-called animal DNA barcoding—is inefficient in species complexes or among cryptic species groups.

Adding to this problem are potential misidentifications in public databases. For example, there are currently five DNA barcode samples for “*Hyphessobrycon*” *santae* (BOLD vouchers: BSB287-10–BSB291-10; NCBI: HM405126.1–HM405130.1), all showing 83–85% similarity with our samples of *P. santae*, *P. terezinhae*, or *P. paiva*. However, the same specimens are listed as “*Hyphessobrycon*” *micropterus* (valid name: *Megalamphodus micropterus*) in SpeciesLink (MCP-Peixes 45159). Upon consulting Professor Roberto Esser dos Reis from PUCRS, where the specimens are deposited, it was possible to confirm through photographs that these specimens likely correspond to *M. micropterus*, displaying the conspicuous black blotch on the dorsal fin characteristic of the genus *Megalamphodus* (Reis, personal communication).

Returning to the context of the potential importance of a history of hybridization and introgression in the evolution of the group, our multilocus phylogenies revealed six well-supported *Psalidodon* clades (Figure 4b,c). However, differences were observed between the two phylogenies, mainly concerning the phylogenetic relationships among the clades. Our phylogenetic network illustrates these discordances, leading us to hypothesize that an intense degree of hybridization and introgression may have occurred during the adaptive radiation of the main clades of the group (Figure 4d). Therefore, it is possible that the phylogenetic relationships among different OTUs of these clades cannot be accurately represented as a bifurcating, two-dimensional tree, regardless of the source or amount of data used. Instead, a phylogenetic network representation is necessary to accommodate the reticulate events that occurred during the evolution of the group [41,92].

Although it is not the focus of this study, we take the opportunity to highlight the possibility of a new *Psalidodon* species in the Paranaíba River hydrographic mesoregion. This region currently hosts two species of this group: the endemic species from Córrego Rita, municipality of Rio Paranaíba/MG, *P. rioparanaibanus*, and its sister species *P.* aff. *paranae* [84]. Both were initially cataloged in the LaGEEvo UFV CRP ichthyological collection as possible specimens of *P. paranae*, but upon analyzing our results, neither belongs to this species. In addition to not clustering with *P. paranae* from the Tietê River in any of our phylogenies (Figure 4), *P. rioparanaibanus* can be distinguished from *P. paranae* and *P.* aff. *paranae* by possessing 34–36 scales along the lateral line (vs. 37–41 scales) and by exhibiting a distinct pattern of light scales interspersed with the dark dorsal scales (de Oliveira, in preparation), whereas *P.* aff. *paranae* can be distinguished from *P. paranae* by having two humeral blotches (vs. one) [14].

## 5. Conclusions

In this study, we aimed to decipher what we term here the “Lütken enigma”. Initially, this enigma refers to the contrast between the null hypothesis that morphotypes M1 and M2 of *“T.” rivularis* represent broad intraspecific morphological variation versus the alternative hypothesis that they correspond to different species. This enigma, however, can be extended to differences in chromosome number and genetic lineages. Here, we demonstrate that by integrating all these sources of data, we were able to conclude that *P. rivularis* sensu lato corresponds not to two or three, but likely five distinct OTUs, whose speciation is the result of a combination of multiple evolutionary factors. Our results also allow us to emphasize the importance of studying and conserving highland environments near the boundaries of different hydrographic mesoregions, such as Rita stream and the Serra da Canastra National Park, as these are conducive to endemism of species within the *P. scabripinnis* complex (here, exemplified by *P. rioparanaibanus* and *P. velhochico*). Furthermore, we stress the importance of preserving small tributaries, which are often neglected but are in fact complex and biodiverse environments, as evidenced here by several cases of sympatry among different OTUs of *P. rivularis* sensu lato.

## Figures and Tables

**Table 1:** Assembled mitochondrial genomes. For the two libraries retrieved from NCBI SRA, the SRR number is provided as the “Library Source”; for the libraries sequenced in this study, the geographic origin of the sample is indicated as the source. ESF: eastern São Francisco and WSF: western São Francisco.

Sample	Origin of the Library	Software	K-Mer	Seed
*P. correntinus*	SRR11147340	NOVOplasty	39	MW495301.1
*P.* aff. *paranae* Paranaíba	Lava-pés stream, Paranaíba River basin	NOVOplasty	35	KX609386.1
*P. rivularis* M1 Lage	Lage stream, Abaeté River basin	NOVOplasty	39	MT428069.1
*P. rivularis* M1 Bonito	Bonito stream, Borrachudo River basin	NOVOplasty	39	MT428069.1
*P. rivularis* M1 Crico	Crico stream, Paracatu River basin	GetOrganelle	115	MT428069.1
*P. rivularis* M1 Funchal	Funchal river, Indaiá River basin	NOVOplasty	39	MT428069.1
*P. rivularis* M1 Borrachudo	Source of the Borrachudo River, Borrachudo river basin	NOVOplasty	39	MT428069.1
*P. rivularis* M1 Velhas	Source of the das Velhas River, das Velhas River basin	NOVOplasty	39	MT428069.1
*P. rivularis* M1 Cipó	Meeting of the Mascates and Bocaina rivers, das Velhas River basin	NOVOplasty	39	MT428069.1
*P. rivularis* M2 Funchal (WSF)	Funchal river, Indaiá River basin	NOVOplasty	39	MT428070.1
*P. rivularis* M2 Lage (WSF)	Lage stream, Abaeté River basin	NOVOplasty	39	MT428070.1
*P. rivularis* M2 Velhas (ESF)	Source of the das Velhas River, das Velhas River basin	NOVOplasty	39	MT428070.1
*P. rivularis* M2 Cipó (ESF)	Meeting of the Mascates and Bocaina rivers, das Velhas River basin	NOVOplasty	39	MT428070.1
*P. rivularis* M3 Canastra	Rasga Canga Waterfall, São Francisco River basin	NOVOplasty	39	MT428069.1
*P. rivularis* M4 Bonito	Bonito stream, Borrachudo River basin	NOVOplasty	39	MT428070.1
*P. scabripinnis*	SRR9985989	NOVOplasty	35	KX609386.1

**Table 2:** Morphometric measurements used in statistical analyses.

Abbreviation	Measurement	Reference Unit
PD	Pre-dorsal fin distance	% of standard length
PP	Pre-pectoral fin distance	% of standard length
PV	Pre-pelvic fin distance	% of standard length
PA	Pre-anal fin distance	% of standard length
BH	Body height	% of standard length
CPH	Caudal peduncle height	% of standard length
CPL	Caudal peduncle length	% of standard length
AFBL	Anal fin base length	% of standard length
ODFD	Orbit to dorsal-fin origin distance	% of standard length
POL	Postorbital length	% of standard length
HL	Head length	% of standard length
OD	Orbit diameter	% of head length
SnL	Snout length	% of head length
UJL	Upper jaw length	% of head length
CPH2	Caudal peduncle height 2	% of body height
HH	Head height	% of body height

**Table 3:** Chromosome numbers and their types for each of the four morphotypes of *P. rivularis*, separated by the location where they were collected and where it was possible to assemble the karyotypes. M: metacentric chromosomes, SM: submetacentric, ST: subtelocentric, A: acrocentric and Bs: supernumerary chromosomes.

Morphotype	Sub-Basin	Collect Site	Number of Metaphases Analyzed	2*n*	FN	Karyotypes				
						M	SM	ST	A	Bs
1	Abaeté	Lage stream	53	46 + 0-2Bs	84	8	22	8	8	0-2
		Tiros stream	24	46 + 0-1B	82	8	20	8	10	0-1
	Borrachudo	Bonito stream	17	46 + 0-1B	82	8	20	8	10	0-1
		Source of the river	5	46 + 0-1B	82	8	20	8	10	0-1
	Indaiá	Funchal River	30	46 + 0-1B	86	8	24	8	6	0-1
	Paracatu	Crico stream	10	46 + 0-1B	82	8	20	8	10	0-1
2	Abaeté	Lage stream	20	50 + 0-1B	90	8	24	8	10	0-1
	Indaiá	Funchal River	11	50	86	8	20	8	14	0
	Das Velhas	Cipó River	10	50	88	8	22	8	12	0
3	São Francisco	Serra da Canastra	31	50 + 0-1B	84	10	18	6	16	0-1
4	Abaeté	Abaeté Power Plant	26	50	90	8	22	10	10	0
	Borrachudo	Bonito stream	13	50	88	8	20	10	12	0-1

**Table 4:** Characteristics with statistically significant differences (*p* ≤ 0.025) in pairwise comparisons of the Dunn test. Significance threshold was adjusted to α/2 = 0.025. ESF: eastern São Francisco and WSF: western São Francisco.

	Morphotype 1	Morphotype 2 ESF	Morphotype 2 WSF	Morphotype 3
**Morphotype 2 ESF**	PD, PP, PA, BH, CPH, CPL, AFBL, ODFD, POL, HL, SnL, UJL, HH, CPH2, LL	-		
**Morphotype 2 WSF**	PP, PV, PA, BH, CPH, CPL, AFBL, ODFD, POL, HL, OD, HH, CPH2, LL	PD, PP, PV, POL, HL, SnL, HH, CPH2	-	
**Morphotype 3**	PP, BH, CPH, CPL, POL, HL, OD, SnL, HH, CPH2	PD, PP, PA, BH, CPH, CPL, AFBL, POL, HL, OD, SnL, UJL, HH, LL	PP, PV, PA, BH, CPH, CPL, AFBL, POL, HL, OD, SnL, HH, LL	-
**Morphotype 4**	PP, POL, HL, OD, LL	PD, PA, BH, CPL, AFBL, POL, OD, HH, CPH2	PP, PA, BH, CPL, AFBL, POL, HL, OD, HH, CPH2	CPH, HL, HH, CPH2, LL

**Table 5:** Classification of specimens into groups according to the Confusion Matrix generated by Random Forest analysis. ESF: eastern São Francisco and WSF: western São Francisco.

		Reference				
		*P. rivularis* m1	*P. rivularis* m2 ESF	*P. rivularis* m2 WSF	*P. rivularis* m3	*P. rivularis* m4
**Predicted**	***P. rivularis* m1**	100%	0	0	0	0
	***P. rivularis* m2 ESF**	0	95.24%	5.26%	0	0
	***P. rivularis* m2 WSF**	0	2.38%	94.74%	0	0
	***P. rivularis* m3**	0	0	0	100%	0
	***P. rivularis* m4**	0	2.36%	0	0	100%

**Table 6:** Morphometric data of *Psalidodon rivularis* sensu stricto. N = sample number, SD = standard deviation.

	*T. rivularis* Lectotype N = 1	*T. rivularis* Paralectotypes N = 2	Non-Types N = 49	Mean	SD
Standard Length	80.7	36.7–67.8	41.0–84.0	60.57	10.91
	% of standard length				
Predorsal distance	47.08	51.66–52.39	46.14–51.86	49.29	1.57
Prepectoral distance	21.76	21.4–23.52	19.42–27.10	22.83	1.53
Prepelvic distance	44.26	42.28–47.78	43.28–50.23	46.43	1.85
Preanal distance	63.94	64.19–65.87	60.87–70.50	65.27	1.80
Body height	33.10	30.84–31.12	25.30–32.71	29.06	1.84
Caudal-peduncle height	11.49	9.95–11.48	9.32–14.72	11.85	0.92
Caudal-peduncle length	14.86	12.60–13.58	11.51–22.77	14.68	1.84
Dorsal-fin length	22.48	23.85	16.49–26.03	20.36	1.93
Pectoral-fin length	18.86	19.17	14.06–22.06	18.20	1.99
Pelvic-fin length	13.33	13.65–14.67	10.36–16.52	13.14	1.94
Anal-fin length	13.35	17.03	10.07–17.11	13.60	1.65
Anal-fin base length	22.63	22.83–23.85	16.44–24.17	21.37	1.81
Orbit—dorsal-fin distance	35.51	36.38–37.35	32.74–37.82	35.06	1.53
Head length	24.36	24.70–26.46	21.40–28.67	25.47	1.35
Postorbital length	10.60	9.67–11.08	9.35–13.11	11.27	0.84
	% of body height				
Head height	74.89	66.69–75.34	67.45–83.72	75.03	3.96
Caudal-peduncle height 2	34.72	32.27–36.89	34.06–52.60	40.92	3.99
	% of head length				
Orbit diameter	28.04	28.99–33.91	26.17–39.24	32.07	2.72
Snout length	30.21	23.83–31.88	16.69–30.20	23.80	3.22
Upper jaw length	43.96	39.78–46.96	29.45–47.40	41.16	3.79
Interorbital distance	-	36.48	27.53–39.61	34.13	2.79

**Table 7:** Morphometric data of *Psalidodon santae* comb. nov. N = sample number, SD = standard deviation.

	*H. santae* Syntypes N = 2	*A. turmalinensis* Types N = 20	Non-Types N = 19	Mean	SD
Standard Length	32.5–54.1	30–54.9	35.71–70.1	45.20	7.94
	% of standard length				
Predorsal distance	53.72–55.00	49.38–53.29	48.98–54.15	51.47	1.46
Prepectoral distance	24.82–24.97	18.10–27.73	18.85–27.77	23.65	2.10
Prepelvic distance	47.70–50.34	44.74–49.28	43.34–50.34	47.19	1.82
Preanal distance	63.59–63.78	61.07–65.49	60.83–68.76	63.73	1.79
Body height	33.75–35.47	32.58–38.48	31.15–38.33	34.75	1.63
Caudal-peduncle height	12.42–12.50	12.32–15.46	9.86–14.13	13.04	1.03
Caudal-peduncle length	11.70–13.55	11.28–14.16	9.38–16.25	13.38	1.35
Dorsal-fin length	22.08–22.26	21.70–26.43	18.84–26.06	23.44	1.90
Pectoral-fin length	18.31	18.67–20.79	17.02–22.97	19.72	1.57
Pelvic-fin length	11.96–13.03	13.17–17.60	13.39–17.27	15.57	1.58
Anal-fin length	20.51–21.52	15.21–19.83	13.98–21.30	17.22	2.18
Anal-fin base length	23.74–26.84	23.86–28.45	20.57–26.45	24.89	1.82
Orbit—dorsal-fin distance	37.94–39.46	33.27–38.73	34.24–39.73	36.60	1.62
Head length	26.95–28.15	24.77–28.84	23.05–30.11	26.68	1.45
Postorbital length	11.41–12.36	10.31–14.57	10.67–14.04	11.90	0.91
	% of body height				
Head height	71.75–73.00	65.14–78.79	63.67–77.79	71.04	3.87
Caudal-peduncle height 2	35.02–37.05	34.35–46.14	29.39–41.35	37.58	3.11
	% of head length				
Orbit diameter	29.52–32.98	29.57–39.06	24.37–35.41	33.08	3.20
Snout length	24.69–26.55	15.60–24.63	18.42–27.34	22.15	2.53
Upper jaw length	45.40–48.13	30.26–43.72	32.52–43.96	38.11	3.97
Interorbital distance	32.42–34.02	31.91–42.87	30.92–38.56	35.90	3.00

**Table 8:** Morphometric data of *Psalidodon terezinhae* sp. nov., *Psalidodon velhochico* sp. nov., *Psalidodon paiva* sp. nov. N = sample number, SD = standard deviation.

	*Psalidodon terezinhae* N = 19			*Psalidodon velhochico* N = 16			*Psalidodon paiva* N = 11		
	Holotype	Amplitude	Mean/SD	Holotype	Amplitude	Mean/SD	Holotype	Amplitude	Mean/SD
Standard Length	53.0	38.0–59.0	47.87/6.05	77.0	49.0–89.0	69.03/12.13	62.0	31.5–77.0	53.32/16.67
	% of standard length								
Predorsal distance	49.28	47.26–53.41	50.15/1.41	49.63	47.60–51.43	49.80/0.80	51.66	48.09–51.66	49.66/0.89
Prepectoral distance	21.74	14.72–23.80	20.69/2.20	22.26	22.26–28.16	25.72/1.86	22.16	22.16–27.47	24.51/1.99
Prepelvic distance	44.70	41.93–49.34	45.30/1.90	45.98	43.90–49.19	46.88/1.57	48.15	42.55–48.15	45.94/1.63
Preanal distance	63.57	59.50–69.79	63.66/2.32	65.25	64.17–67.69	65.83/0.96	66.01	62.88–68.21	66.28/1.56
Body height	38.90	33.97–38.90	35.88/1.55	33.40	28.06–33.40	30.87/1.48	28.64	28.64–32.66	30.90/1.34
Caudal-peduncle height	14.56	11.23–14.56	12.51/0.78	11.20	9.10–12.71	11.03/0.92	12.02	11.41–13.16	12.42/0.46
Caudal-peduncle length	12.49	10.47–15.78	12.72/1.32	16.00	13.54–18.15	16.26/1.07	15.98	13.30–18.60	15.64/1.56
Dorsal-fin length	21.98	20.32–27.49	24.34/2.12	17.57	15.79–21.39	18.65/1.77	-	20.49–29.47	25.33/3.26
Pectoral-fin length	20.14	17.67–20.83	19.36/1.02	18.63	17.98–19.97	18.75/0.63	18.43	15.97–19.09	17.15/1.69
Pelvic-fin length	13.33	11.75–17.68	14.69/1.58	16.38	12.86–16.44	14.78/1.33	13.80	12.72–18.41	14.67/3.24
Anal-fin length	13.95	7.65–16.96	13.97/2.44	11.57	7.75–16.03	12.89/2.10	-	12.84–18.78	15.53/2.69
Anal-fin base length	27.68	22.48–29.16	26.17/1.83	21.51	16.21–23.26	19.96/1.74	18.88	15.51–21.64	19.34/1.85
Orbit—dorsal-fin distance	36.04	33.36–37.99	36.12/1.26	37.10	34.41–37.10	35.77/0.75	39.70	33.42–39.70	35.94/2.00
Head length	23.82	21.60–26.19	23.92/1.26	27.23	26.84–31.93	29.07/1.59	25.67	24.42–29.92	26.97/1.85
Postorbital length	10.46	8.60–11.00	09.93/0.71	14.80	13.80–17.25	15.10/0.98	13.71	12.24–14.52	13.25/0.69
	% of body height								
Head height	59.24	59.24–69.30	64.18/2.35	78.61	75.44–87.22	80.98/3.72	79.22	68.34–79.25	75.59/3.81
Caudal-peduncle height 2	37.43	31.34–38.01	34.87/1.89	33.54	30.82–41.84	35.76/2.83	41.97	35.28–43.16	40.27/2.37
	% of head length								
Orbit diameter	39.11	29.20–39.11	34.22/2.01	23.83	23.22–33.18	28.06/2.88	25.12	23.27–34.72	27.82/3.76
Snout length	16.47	16.47–30.71	24.30/3.07	22.19	15.75–24.45	20.33/2.51	21.47	18.09–26.67	22.62/2.56
Upper jaw length	33.84	33.84–45.69	39.89/2.90	39.02	35.28–48.40	40.36/3.00	43.05	33.80–43.47	39.32/3.35
Interorbital distance	39.61	30.07–39.61	34.69/3.18	33.39	27.58–37.10	32.62/2.86	34.56	29.47–39.38	34.42/2.82

## Data Availability

The genomic libraries generated in this study have been deposited in the NCBI Sequence Read Archive (SRA) and will be available upon publication under BioProject accession number PRJNA1335291. The mitochondrial genomes generated in this study have been submitted to GenBank and will be publicly available upon publication under accession numbers PX440285-PX440298 and BK075098-BK075099.

## Supplementary Materials

The following supporting information can be downloaded at: https://www.mdpi.com/article/10.3390/biology14121793/s1, Material S1: Material analyzed. Analyzed species: *Psalidodon rivularis*, *Psalidodon santae* comb. nov., *Psalidodon terezinhae* sp. nov., *Psalidodon velhochico* sp. nov., *Psalidodon paiva* sp. nov., “*Astyanax turmalinensis*”, *Psalidodon* aff. *paranae*, *Psalidodon rioparanaibanus*, *Psalidodon fasciatus*, and *Astyanax lacustris*; Material S2: Protocol adapted from Bertollo (1978) for obtaining mitotic chromosomes in characins; Material S3: DNA extraction protocol from fish tissues using the Quick-DNA/RNA Viral MagBead kit (Zymo); Material S4: Protocol for using short-read genomic data in phylogenomic analyses with orthologous protein coding genes, adapted from Roncoroni & Gallone (2022); Spreadsheet S5: Features table containing the start and end coordinates of each mitochondrial feature, alongside with their respective length in base pair (bp). Figure S1: Time-calibrated phylogeny inferred using the LSD2 method as implemented in IQ-TREE, based on the 90% matrix. The timescale is shown in millions of years (Mya). Numbers above the branches indicate estimated divergence times, and numbers below the branches represent the 95% confidence intervals. The variance parameter was set to −b = 0.0115135, and the relaxed-clock standard deviation to −q = 0.2.

## Abbreviations

The following abbreviations are used in this manuscript:

M1	Morphotype 1 of *Psalidodon rivularis*
M2	Morphotype 2 of *Psalidodon rivularis*
M3	Morphotype 3 of *Psalidodon rivularis*
M4	Morphotype 4 of *Psalidodon rivularis*
mtDNA	Mitochondrial DNA
NGS	Next-Generation Sequencing
LaGEEvo	Laboratory of Ecological and Evolutionary Genetics
UFV–CRP	Federal University of Viçosa–Rio Paranaíba Campus
DZUFMG	Department of Zoology at the Federal University of Minas Gerais
CT Scan	Computed Tomography scan
SISBIO	Biodiversity Authorization and Information System
CONCEA	National Council for Animal Control and Experimentation
MCTI	Ministry of Science, Technology, Innovations and Communications
CEUA	Ethics Committee on Animal Use
PCGs	Protein-coding genes
rRNA	Ribosomal RNA
tRNA	Transfer RNA
NCBI	National Center for Biotechnology Information
SRA	Sequence Read Archive
OTUs	Operational taxonomic units
PCA	Principal Component Analysis
LL	Scales along the longitudinal series/lateral line
SL	Standard length
BH	Body Height/depth
AFBL	Anal fin base length
HL	Head length
HH	Head Height/depth
OOL	Orbit–opercle length
POL	Postorbital length
CI	Confidence Interval
Mya	Millions of years ago
SD	Standard deviation
WSF	West of São Francisco River basin
ESF	East of São Francisco River basin
