# Supplementary material for: Unraveling a 150-Year-Old Enigma: Psalidodon rivularis (Acestrorhamphidae: Acestrorhampinae), a Species Complex or a Polymorphic Species?
Source: Biology (Basel). 2025 Dec 16;14(12):1793. doi: 10.3390/biology14121793 (PMC12730566; doi:10.3390/biology14121793)
Supplement: Supplementary file 1 [file biology-14-01793-s001.zip › Supplementary Material S1 .pdf]

Supplementary Material S1 – Material analyzed. Analyzed species: *Psalidodon rivularis*, *Psalidodon santae* comb. nov., *Psalidodon terezinhae* sp. nov., *Psalidodon velhochico* sp. nov., *Psalidodon paiva* sp. nov., "*Astyanax turmalinensis*", *Psalidodon aff. paranae*, *Psalidodon rioparanaibanus*, *Psalidodon fasciatus*, and *Astyanax lacustris*.

Material	Species	Nº of individuals	Size (min – max)	Local	Date	Collectors
			**Material collected during this work**			
LaGEEvo-27	*P. terezinhae*	1	53mm	Lage stream, Arapuá/MG, 19°1'25.18"S 46°6'18.74"O	2022-11-05	I.H.R. Oliveira, P.M. de Assis, T. da S. Santos
LaGEEvo-28	*P. velhochico*	1	77mm	Upper part of Rasga Canga waterfall, Serra da Canastra National Park/MG, 20°10'39.13"S 46°33'33.68"O	2023-07-29	I.H.R. Oliveira, I.B. da Silva, P.M. de Assis, L.G.P. Pimentel, R.A.S. Soares, T. da S. Ramos, J. Godoy, S.S.N. Pereira
LaGEEvo-30	*P. rivularis*	12	45mm – 63.5mm	Lage stream, Arapuá/MG, 19°1'25.18"S 46°6'18.74"O	2022-11-05	I.H.R. Oliveira, P.M. de Assis, T. da S. Santos
LaGEEvo-31	*P. terezinhae*	22	38mm – 54mm	Lage stream, Arapuá/MG, 19°1'25.18"S 46°6'18.74"O	2022-11-05	I.H.R. Oliveira, P.M. de Assis, T. da S. Santos
LaGEEvo-32	*P. santae*	21	31mm – 49mm	das Velhas river, Ouro Preto/MG 20°20'38.4"S 43°29'58.4"O	2023-09-15	I.H.R. Oliveira, I.B. da Silva, P.M. de Assis, L.G.P. Pimentel,

						R. Pasa
LaGEEvo-33	*P. rivularis*	4	46mm – 69.5mm	das Velhas river,<br>Ouro Preto/MG<br>20°20'38.4"S<br>43°29'58.4"O	2023-09-15	I.H.R. Oliveira,<br>I.B. da Silva,<br>P.M. de Assis,<br>L.G.P. Pimentel,<br>R. Pasa
LaGEEvo-34	*P. rivularis*	3	53mm – 64mm	Borrachudo river,<br>Matutina/MG<br>19°13'02.6"S<br>45°55'58.2"O	2023-03-08	I.H.R. Oliveira,<br>P.M. de Assis,<br>R.A.S. Soares,<br>R. Pasa
LaGEEvo-35	*P. terezinhae*	6	35mm – 54mm	Funchal river,<br>São Gotardo/MG<br>19°24'9.54"S<br>46°0'4.61"O	2023-12-02	I.H.R. Oliveira,<br>I.B. da Silva,<br>P.M. de Assis,<br>L.G.P. Pimentel,<br>R. Pasa
LaGEEvo-36	*P. rivularis*	4	41mm – 46mm	Funchal river,<br>São Gotardo/MG<br>19°24'9.54"S<br>46°0'4.61"O	2023-12-02	I.H.R. Oliveira,<br>I.B. da Silva,<br>P.M. de Assis,<br>L.G.P. Pimentel,<br>R. Pasa
LaGEEvo-48	*P. rivularis*	23	47mm – 84mm	meeting of Mascates<br>and Bocaina rivers,<br>Serra do Cipó<br>National Park/MG,<br>19°20'49.68"S<br>43°36'20.42"O	2023-10-09	I.H.R. Oliveira,<br>I. B. da Silva,<br>P.M. de Assis,<br>L.G.P. Pimentel,<br>R.A.S. Soares,<br>G.F. da Fonseca,<br>G.F.Matos,<br>V.G. de Miranda,<br>B. Alonso

LaGEEvo-49	*P. santae*	8	41mm – 59.5mm	meeting of Mascates and Bocaina rivers, Serra do Cipó National Park/MG, 19°20'49.68"S 43°36'20.42"O	2023-10-09	I.H.R. Oliveira, I. B. da Silva, P.M. de Assis, L.G.P. Pimentel, R.A.S. Soares, G.F. da Fonseca, G.F.Matos, V.G. de Miranda, B. Alonso
LaGEEvo-50	*P. velhochico*	15	49mm – 89mm	Upper part of Rasga Canga waterfall, Serra da Canastra National Park/MG, 20°10'39.13"S 46°33'33.68"O	2023-07-29	I.H.R. Oliveira, I.B. da Silva, P.M. de Assis, L.G.P. Pimentel, R.A.S. Soares, T. da S. Ramos, J. Godoy, S.S.N. Pereira
LaGEEvo-51	*P. velhochico*	34	43mm – 83mm	Casca d' Anta waterfall, Serra da Canastra National Park/MG, 20°18'2.24"S 46°31'18.99"O	2023-07-28	I.H.R. Oliveira, I.B. da Silva, P.M. de Assis, L.G.P. Pimentel, R.A.S. Soares, T. da S. Ramos, J. Godoy, S.S.N. Pereira
LaGEEvo-52	*P. velhochico*	34	40mm – 81mm	Historical source of the São Francisco river, Serra da Canastra National Park/MG,	2023-07-28	I.H.R. Oliveira, I.B. da Silva, P.M. de Assis, L.G.P. Pimentel, R.A.S. Soares,

				20°14'4.79"S 46°26'29.38"O		T. da S. Ramos, J. Godoy, S.S.N. Pereira
LaGEEvo-53	*P. velhochico*	23	50mm – 93mm	Lower part of Rasga Canga waterfall, Serra da Canastra National Park/MG, 20°10'39.13"S 46°33'33.68"O	2023-07-29	I.H.R. Oliveira, I.B. da Silva, P.M. de Assis, L.G.P. Pimentel, R.A.S. Soares, T. da S. Ramos, J. Godoy, S.S.N. Pereira
LaGEEvo-58	*P. santae*	4	56mm – 72mm	Bandeirinhas Cânion, Serra do Cipó National Park/MG, 19°25'8.33"S 43°34'12.37"O	2023-10-11	I.H.R. Oliveira, I. B. da Silva, P.M. de Assis, L.G.P. Pimentel, R.A.S. Soares, G.F. da Fonseca, G.F.Matos, V.G. de Miranda, B. Alonso, R. Pasa
LaGEEvo-60	*P. rivularis*	11	64mm – 99mm	Farofa waterfall trail, Serra do Cipó National Park/MG, 19°23'6.52"S 43°35'12.28"O	2023-10-10	I.H.R. Oliveira, I. B. da Silva, P.M. de Assis, L.G.P. Pimentel, R.A.S. Soares, G.F. da Fonseca, G.F.Matos, V.G. de Miranda, B. Alonso,

						R. Pasa
LaGEEvo-62	*P. santae*	1	63mm	Farofa waterfall trail, Serra do Cipó National Park/MG, 19°23'6.52"S 43°35'12.28"O	2023-10-10	I.H.R. Oliveira, I. B. da Silva, P.M. de Assis, L.G.P. Pimentel, R.A.S. Soares, G.F. da Fonseca, G.F.Matos, V.G. de Miranda, B. Alonso, R. Pasa
LaGEEvo-65	*A. lacustris*	2	69mm – 78.5mm	Retiro de Baixo stream, Lagoa da Prata/MG, 20° 0'15.75"S 45°30'42.13"O	2023-05-06	I.H.R. Oliveira, I.B. da Silva, R.A.S. Soares, L. da C. de Santos
LaGEEvo-67	*P. fasciatus*	1	98mm	Retiro de Baixo stream, Lagoa da Prata/MG, 20° 0'15.75"S 45°30'42.13"O	2023-05-06	I.H.R. Oliveira, I.B. da Silva, R.A.S. Soares, L. da C. de Santos
LaGEEvo-69	*P. fasciatus*	7	38.6mm – 89mm	Santana river, Lagoa da Prata/MG, 20° 4'32.68"S 45°32'52.10"O	2023-05-06	I.H.R. Oliveira, I.B. da Silva, R.A.S. Soares, L. da C. de Santos
LaGEEvo-71	*A. lacustris*	12	49mm – 59mm	Abaeté Power Plant Dam lagoon, Rio Paranaíba/MG, 19°12'35.69"S 46° 6'33.79"O	2022-10-01	I.H.R. Oliveira, P.M. de Assis, L.G.P. Pimentel, R. A. S. Soares

LaGEEvo-74	*P. rivularis*	11	56mm – 87.5mm	Confusão stream, São Gotardo/MG, 19°20'21.89"S 46° 6'21.38"O	2024-11-11	I.H.R. Oliveira, P.M. de Assis, W. Cléber
**Material deposited in the UFV-CRP Vertebrate Collection**						
LaGEEvo-12	*P. rioparanaibanus*	1	84mm	Rita stream, Rio Paranaíba/MG, 19°11'15.77"S 46°14'10.24"O	2017-08-21	M.A. da Silva, I.B. da Silva
LaGEEvo-13	*P. rioparanaibanus*	8	43mm – 84mm	Rita stream, Rio Paranaíba/MG, 19°11'15.77"S 46°14'10.24"O	2018-09-23	I.H.R. Oliveira, I.B. da Silva, R.L.Oliveira, G. Leles, T. Castaño
LaGEEvo-14	*P. rioparanaibanus*	4	40mm – 84mm	Rita stream, Rio Paranaíba/MG, 19°11'15.77"S 46°14'10.24"O	2017-08-21	M.A. da Silva, I.B. da Silva
LaGEEvo-29	*P. paiva*	1	62mm	Bonito stream, Tiros/MG, 18°48'44.7"S 45°45'52.2"O	2010-07-20	P. Penteado, Denis, Gabriel, Rafael
LaGEEvo-37	*P. rivularis*	2	47mm – 56mm	Funchal river, São Gotardo/MG, 19°24'9.54"S 46°0'4.61"O	2019-05-04	I.H.R. Oliveira, I.B. da Silva, L. Fainé, J. Godoy
LaGEEvo-38	*P. terezinhae*	1	59mm	Funchal river, São Gotardo/MG, 19°24'9.54"S 46°0'4.61"O	2019-05-04	I.H.R. Oliveira, I.B. da Silva, L. Fainé, J. Godoy

LaGEEvo-39	*P. paiva*	5	52mm – 77mm	Bonito stream, Tiros/MG, 18°48'44.7"S 45°45'52.2"O	2010-07-20	P. Penteado, Denis, Gabriel, Rafael
LaGEEvo-40	*P. rivularis*	12	64mm – 86mm	Bonito stream, Tiros/MG, 18°48'44.7"S 45°45'52.2"O	2010-07-20	P. Penteado, Denis, Gabriel, Rafael
LaGEEvo-41	*P. rivularis*	17	48mm – 79.5mm	Lage stream, Arapuá/MG, 19°1’25.18”S 46°6’18.74”O	2010-07-23	P. Penteado, D. Reis, Denis, Paloma, Wanessa
LaGEEvo-42	*P. terezinhae*	15	36.5mm – 49mm	Lage stream, Arapuá/MG, 19°1’25.18”S 46°6’18.74”O	2010-07-23	P. Penteado, D. Reis, Denis, Paloma, Wanessa
LaGEEvo-43	*P. rivularis*	9	43mm – 52mm	Crico stream, Presidente Olegário/MG, 18°18'44.36"S 46° 5'44.46"O	2019-04-06	I.B. da Silva, M.L.C.B. de Campos, V. Augusto, S.V. Resende
LaGEEvo-44	*P. rivularis*	4	43mm – 52mm	Bandeirinhas cânion, Serra do Cipó National Park, 19°25'8.33"S 43°34'12.37"O	2017-10	M.L.C.B. de Campos, R.R. Rocha, S.V. Resende, F. Sassi
LaGEEvo-45	*P. santae*	9	43mm – 53mm	Bandeirinhas cânion,	2017-10	M.L.C.B. de Campos,

				Serra do Cipó National Park, 19°25'8.33"S 43°34'12.37"O		R.R. Rocha, S.V. Resende, F. Sassi
LaGEEvo-46	*P. rivularis*	9	42mm – 59mm	Bandeirinhas stream, Serra do Cipó National Park, 19°24'32.65"S 43°34'35.31"O	2017-10	M.L.C.B. de Campos, R.R. Rocha, S.V. Resende, F. Sassi
LaGEEvo-47	*P. santae*	6	42mm – 49mm	Bandeirinhas stream, Serra do Cipó National Park, 19°24'32.65"S 43°34'35.31"O	2017-10	M.L.C.B. de Campos, R.R. Rocha, S.V. Resende, F. Sassi
LaGEEvo-54	*P. paiva*	5	42mm – 72mm	Abaeté Power Plant Dam lagoon, Rio Paranaíba/MG, 19°12'35.69"S 46° 6'33.79"O	2012-07-17	W. Lopes-Silva, C.H.M. Fernandes, M.A. da Silva, A.C.M. Fernandes
LaGEEvo-55	*P. paiva*	10	31.5mm – 44mm	Abaeté Power Plant Dam lagoon, Rio Paranaíba/MG, 19°12'35.69"S 46° 6'33.79"O	2017-07-07	I.H.R. Oliveira, I.B. da Silva, M.A. da Silva
LaGEEvo-56	*P. rivularis*	14	48mm – 69mm	Tiros stream, Tiros/MG, 18°56'34.08"S 45°56'18.20"O	2010-07-20	P. Penteado, Denis, Gabriel, Rafael
LaGEEvo-57	*P. paiva*	4	57.5mm – 67.5mm	Tiros stream, Tiros/MG,	2010-2012	-

				18°56'34.08"S 45°56'18.20"O		
LaGEEvo-59	*P. rivularis*	3	50mm – 65mm	Funchal river, São Gotardo/MG, 19°24'9.54"S 46°0'4.61"O	2018-05-12	I.H.R. Oliveira, I.B. da Silva, G. Bork, L. Fernandes, V. Augusto
LaGEEvo-63	*P. rivularis*	4	50mm – 81mm	Vereda Grande river, Três Marias/MG, 18°19'18.62"S 45° 6'32.80"O	2017-04-18	R. de M. Alves; R.R. Rocha; M.A. da Silva; S.V. Resende
LaGEEvo-64	*P. rivularis*	5	55mm – 62mm	Vereda Grande river, Três Marias/MG, 18°19'18.62"S 45° 6'32.80"O	2010-2012	-
LaGEEvo-66	*A. lacustris*	3	69mm – 79mm	Teixeira stream, Augusto de Lima/MG, 17º58'58.53"S 44º4'26.566"O	2024-07	V.G. de Miranda
LaGEEvo-68	*P. fasciatus*	3	68mm – 83mm	Teixeira stream, Augusto de Lima/MG, 17º58'58.53"S 44º4'26.566"O	2024-07	V.G. de Miranda
LaGEEvo-70	*P. fasciatus*	1	64mm	Vereda Grande river, Três Marias/MG, 18°19'18.62"S 45° 6'32.80"O	2017-04-18	R. de M. Alves; R.R. Rocha; M.A. da Silva; S.V. Resende
LaGEEvo-72	*P. paranae*	7	53.5mm – 77.5mm	Lava-Pés stream, Rio Paranaíba/MG,	2015-11	M.L.C.B. de Campos, F. Sassi,

				19°11'41.22"S 46°15'7.32"O		T. Lunardi, R. Pereira, M. Trevisanuto
LaGEEvo-73	*P. paranae*	3	77mm – 83mm	Lava-Pés stream, Rio Paranaíba/MG, 19°11'41.22"S 46°15'7.32"O	2015-11	M.L.C.B. de Campos, F. Sassi, T. Lunardi, R. Pereira, M. Trevisanuto
LaGEEvo-75	*P. rivularis*	4	56mm – 87.5mm	Espinha stream, Tiros/MG 19° 2'47.18"S 46° 1'12.93"O	2016-07-24	M.L.C.B. de Campos, M.A. da Silva, S.V. Resende, R. Pasa
LaGEEvo-76	*P. paiva*	9	37mm – 45mm	Crico stream, Presidente Olegário/MG, 18°18'44.36"S 46° 5'44.46"O	2019-04-06	I.B. da Silva, M.L.C.B. de Campos, V. Augusto, S.V. Resende
**Material depositado no Departamento de Zoologia da UFMG**						
DZUFMG 005	*"A. turmalinensis"*	1	48.2mm	Córrego Divisão, Turmalina/MG, 17°07'00.00"S 42°57'00.00"O	Mai/1989	V. Vono
DZUFMG 009	*"A. turmalinensis"*	19	33.5mm – 54.9mm	Córrego Divisão, Turmalina/MG, 17°07'00.00"S 42°57'00.00"O	Ago/1989	V. Vono
**Material (fotos and notes) by C.A.M. Oliveira (2017)**						
ZMUC	*P. santae*	2	37.5mm – 60.1mm	das Velhas river,	1847-1854	J.T. Reinhardt

				Lagoa Santa/MG		
USNM 44960	*P. rivularis*	2	37.6mm – 67.8mm	das Velhas river, Lagoa Santa/MG	1847-1854	J.T. Reinhardt
USNM 55652	*P. santae*	2	32.5mm – 54.1mm	das Velhas river, Lagoa Santa/MG	1847-1854	J.T. Reinhardt
MCZ 20874	*P. rivularis*	1	67.61mm	das Velhas river, Lagoa Santa/MG, 19°27'26''S 44°14'30''O	1865-07	G. Sceva & Thayer Expedition
**Material (photographs) website of the biological collections of the Natural History Museum of Denmark**						
ZMUC P241289	*P. rivularis*	1	80.7mm	das Velhas river, Lagoa Santa/MG	1847-1854	J.T. Reinhardt
ZMUC P241329	*A. lacustris*	1	51.12mm	das Velhas river, Lagoa Santa/MG	1847-1854	J.T. Reinhardt
ZMUC P241291	*P. fasciatus*	1	86.5mm	das Velhas river, Lagoa Santa/MG	1847-1854	J.T. Reinhardt
