# Supplementary material for: Unraveling a 150-Year-Old Enigma: Psalidodon rivularis (Acestrorhamphidae: Acestrorhampinae), a Species Complex or a Polymorphic Species?
Source: Biology (Basel). 2025 Dec 16;14(12):1793. doi: 10.3390/biology14121793 (PMC12730566; doi:10.3390/biology14121793)
Supplement: Supplementary file 1 [file biology-14-01793-s001.zip › Supplementary Material S2 .pdf]

Supplementary Material S2 – Protocol adapted from Bertollo [1] for obtaining mitotic chromosomes in characins.

1. Pre-preparation

1.1 It is recommended to acclimate the fish in the laboratory before preparation, keeping the specimens in aquariums under controlled temperature and conditions. The acclimation period may vary between species; in our experience, the best results were obtained after 96 hours for *Astyanax lacustris* and 120 hours for *Psalidodon rivularis*.

1.2 An immunological induction technique is also recommended approximately 24 to 48 hours before euthanasia, such as intraperitoneal induction of 1ml of Broncho-Vaxom® per 50g of body weight [2].

1.3 Prior to preparation, euthanize the specimens by immersion in 1% Eugenol diluted in 1 L of water, following Normative Resolution No. 37 of the National Council for the Control of Animal Experimentation of the Ministry of Science, Technology, Innovations and Communications (CONCEA – MCTI).

1.4 While the fish are being euthanized, separate and prepare the necessary reagents: place the hypotonic solution (0.2 M KCl) and culture medium (Leibowitz, RPMI, etc.) in an incubator at 37 °C. Prepare the fixative (Methanol 3:1 Acetic Acid) and store it in the freezer (60 mL is sufficient for four specimens with leftovers).

1.5 Organize the necessary tools: surgical forceps and scissors, containers for the culture medium, syringes, suspension tubes, pipettes, and Eppendorf tubes (two per specimen if tissue will be collected for DNA extraction; store the tissue in absolute ethanol). Important: label the containers, suspension tubes, pipettes, and Eppendorf tubes for each fish.

2. Pre-fixation

2.1 Add 6 mL of culture medium to the containers.

2.2 Open the specimen by making an incision from the cloaca to the opercular region. Remove the anterior kidney, posterior kidney, and spleen, and place them in the culture medium. Record the sex whenever possible by directly observing the gonads.

2.3 Dissociate the tissue using syringes, usually for 5 to 15 minutes, until the tissue is completely dissociated. Remove any fibrous skin fragments with the help of forceps.

2.4 Transfer the dissociated material to centrifuge tubes, add 200 μL of 0.0125% colchicine. Resuspend and incubate at 37 °C for 15–20 minutes.

2.5 Resuspend the material again and centrifuge at 1000×g for 10 minutes.

2.6 Carefully remove the supernatant by decanting it from the side opposite to where the material is adhered to the tube wall, into a discard beaker. Use a paper towel to absorb the remaining liquid by capillarity.

2.7 Add 6 mL of KCl and resuspend. Incubate at 37 °C for 25 minutes.

2.8 After hypotonic treatment, remove any remaining tissue fragments in suspension using pipettes.

2.9 Add 10 drops of fixative and resuspend (from this step forward, avoid bubble formation as much as possible). Centrifuge at 1000×g for 10 minutes. Remember to return the fixative to the freezer.

3. Fixation

3.1 Carefully remove the supernatant by decanting it from the side opposite to where the material is adhered to the tube wall, into a discard beaker. Use a paper towel to absorb the remaining liquid by capillarity.

3.2 Add 6ml of fixative, dripping down the side of the tube where most of the material is stuck, and resuspend, avoiding bubble formation. Centrifuge at 1000xg for 10 minutes. Remember to return the fixative to the freezer.

3.3 Repeat the above steps at least one more time. If you notice that two fixations are not enough to ensure good chromosome quality, a third fixation can be applied by adjusting the maximum amount of fixative.

3.4 After the last fixation, carefully remove the supernatant, pouring it, on the opposite side to where the material is stuck to the wall of the tube, into a beaker for disposal, using a paper towel to absorb the remaining liquid by capillarity.

3.5 Add 1 mL of fixative slowly down the tube wall where most of the material is adhered. Resuspend while avoiding bubbles and transfer the material to an Eppendorf tube.

4. Referências

1. Bertollo, L.A.C. Cytotaxonomy Considerations on *Hoplias Lacerdae* (Pisces: Erythrynidae). *Brazil. J. Genet.* **1978**, *2*, 17–37.
2. Molina, W.F.; Alves, D.E.O.; Araújo, W.C.; Martinez, P.A.; Silva, M.F.M.; Costa, G. Performance of Human Immunostimulating Agents in the Improvement of Fish Cytogenetic Preparations. *Genet Mol Res* **2010**, *9*, 1807–1814.
