# Supplementary material for: Unraveling a 150-Year-Old Enigma: Psalidodon rivularis (Acestrorhamphidae: Acestrorhampinae), a Species Complex or a Polymorphic Species?
Source: Biology (Basel). 2025 Dec 16;14(12):1793. doi: 10.3390/biology14121793 (PMC12730566; doi:10.3390/biology14121793)
Supplement: Supplementary file 1 [file biology-14-01793-s001.zip › Supplementary Material S3.pdf]

Supplementary Material S3 - DNA extraction protocol from fish tissues using the Quick-DNA/RNA Viral MagBead kit (Zymo).

1. Using surgical forceps and scissors, remove small pieces of tissue (approximately 20 mg) and place them in an open Eppendorf tube to allow the alcohol in the tissue to evaporate.

2. Grind the dry tissue using a pestle.

3. Add 200 µL of DNA/RNA Shield (1X), letting the liquid run down the pestle to dislodge any remaining tissue fragments.

4. Add 15 µL of Proteinase K.

5. Incubate the material in a dry bath at 56 °C for 3–4 hours. We recommend vortexing the sample before incubation and every hour during incubation.

6. If the tissue has been fully digested, prepare the plate (up to 16 samples per plate) for automated extraction using a Loccus Extracta 32 device as follows:

**Columns:**	**1 e 7**	**2 e 8**	**3 e 9**	**4, 5, 10 e 11**	**6 e 12**
	400uL of Buffer<br>205uL of sample<br>10uL of Beads	250uL of Wash 1	250uL of Wash2	250uL of Ethanol PA	50uL of DNA/RNA-free Water

7. Program a protocol in the extractor as follows:

**Step**	**Pit**	**Name**	**Agitation (Min)**	**Magnet. (Seg)**	**Wait (Min)**	**Volume (uL)**	**Speed (1-10)**	**Temp. (°C)**
1	1	Lise	10	120	0	615	5	OFF
2	2	Wash	5	60	0	250	5	OFF
3	3	Wash	2	60	0	250	5	OFF
4	4	Ethanol	2	60	0	250	5	OFF
5	5	Ethanol	2	60	0	250	5	OFF
6	6	Dry	0	0	10	200	5	OFF
7	6	Elution	3	240	0	50	3	OFF
8	1	Discard	1	0	0	835	5	OFF
