# Supplementary material for: Unraveling a 150-Year-Old Enigma: Psalidodon rivularis (Acestrorhamphidae: Acestrorhampinae), a Species Complex or a Polymorphic Species?
Source: Biology (Basel). 2025 Dec 16;14(12):1793. doi: 10.3390/biology14121793 (PMC12730566; doi:10.3390/biology14121793)
Supplement: Supplementary file 1 [file biology-14-01793-s001.zip › Supplementary Material S4.pdf]

Supplementary Material S4 - Protocol for using short-read genomic data in phylogenomic analyses with orthologous protein coding genes, adapted from Roncoroni & Gallone [1]**:**

1. **Import the raw reads into Galaxy Europe platform** [2]**.**

2. **Filter the raw reads with Trimmomatic tool** [3]**:**

   Single-end or paired-end reads?

   Paired-end

   Input FASTQ file (R1/first of pair) *

   Select your data (R1 or forward reads)

   Input FASTQ file (R2/second of pair) *

   Select your data (R2 or reverse reads)

   Perform initial ILLUMINACLIP step?

   yes

   Adapter sequences to use *

   TruSeq3 (paired-end, for MiSeq and HiSeq)

   *leave other options in default mode

3. **Quality evaluation with FastQC** [4]

   Raw read data from your current history *

   Select your data (R1 paired and R2 paired)

   *leave other options in default mode

4. **Assembly the short reads into genome scaffolds with MEGAHIT tool** [5]**:**

   Select your input option

   Paired-end

   Mate 1 input reads *

   Select your data (R1 paired)

Mate 2 input reads *

Select your data (R2 paired)

*leave other options in default mode

5. **Removing isoforms with cd-hit tool** [6]**:**

Sequences to cluster/compare *

Select your data (output of MEGAHIT)

Sequence type?

Nucleotide

Sequence identity threshold *

0,95

Compare both strands?

Yes

*leave other options in default mode

6. **Assembly quality evaluation with Fasta Statistics tool** [7]**:**

FASTA or Multi-FASTA file *

Select your data (Representative Sequences output of ch-hit)

*leave other options in default mode

7. **Assembly quality evaluation with Busco tool** [8]**:**

Sequences to analyze *

Select your data (Representative Sequences output of ch-hit)

Lineage data source

Download Lineage Data

Mode

Genomic assemblies (DNA)

Auto-detect or select lineage?

Select lineage

Lineage *

Actinopterygii (Adjust according to the study group)

Which outputs should be generated

short summary text

summary image

*leave other options in default mode

8. Rename fasta headers with Replace Text (Necessary to RepeatMasker tool) [9]**:**

File to process *

Select your data (Representative Sequences output of ch-hit)

1: Replacement

(>[^]+).+

Replace with:

\1

9. Masking repeated regions with RepeatMasker tool [10]**:**

Genomic DNA *

Select your data (Renamed fasta output)

Repeat library source

DFam (curated only, bundle with RepeatMasker)

Select species name from a list?

Yes

Species *

Danio (zebra fish) (Adjust according to the study group)

Output annotation of repeats in GFF format

yes

Perform softmasking instead of hardmasking

yes

*leave other options in default mode

10. Filtering bad contigs with Filter FASTA on the headers and/or the sequences:

FASTA sequences *

Select your data (Masked sequences output of RepeatMasker)

Criteria for filtering on the sequences

Regular expression on the sequences

Regular expression pattern the sequence should match *

^(?=.*A)(?=.*T)(?=.*C)(?=.*G)

*leave other options in default mode

11. Genome annotation with Augustus [11]**:**

Genome Sequence *

Select your data (Filtered sequences output of Filter FASTA tool)

Trainingset

Run Augustus with a predefined trainingset

Model Organism *

Danio rerio (Adjust according to the study group)

Softmasking

yes

GFF formated output

Yes

12. Create a dataset with all protein coding genes multifastas:

Use coding sequences sequences generate by Augustus tool

13. Rename each multifasta within your dataset according to the sample/species name:

14. Rename fasta headers with Regex Find And Replace:

Select lines from *

Select your data (dataset)

Check

Find Regex

>([^]+.+)

Replacement

>#{input_name}_\1

*leave other options in default mode

15. **Keep only sequences from a certain size with Filter FASTA on the headers and/or the sequences:**

FASTA sequences *

Select your data (coding sequences dataset)

Criteria for filtering on the headers

No filtering

Criteria for filtering on the sequences

Sequence length

Minimum length *

300 (Adjust according to your preference)

*leave other options in default mode

16. **Find orthologs with Proteinortho tool** [12]**:**

Select the input fasta files (>2) *

Select your data (renamed dataset)

Similarity comparision algorithm *

NCBI-BLASTN+ (nucleotide sequences)

*leave other options in default mode

17. **Filter information of single copy orthologs presents in all samples with Filter tool:**

Filter

Select your data (orthology-groups file generated by Proteinortho)

With following condition

c1==25* and c2==25*

*This number is related to the number of species in your analysis, in the example above the number of species is 25

18. **Extract single copy orthologs presents in all samples with Proteinortho grab proteins** [12]**:**

Select the input fasta files

Select your data (renamed dataset)

A orthology-groups file *

Select your data (filtered information of orthology-groups file)

*leave other options in default mode.

19. **Rename single copy orthologs with species name with Replace text tool** [9]**:**

File to process

Select your data (extracted single copy orthologs dataset)

Find pattern

>([^_]+_[^_]+).*

Replace with:

>\1

20. **Align genes with MAFFT** [13]**:**

1: Input batch

Sequences to align *

Select your data (renamed single copy orthologs dataset)

Type of sequences

Nucleic acids

MAFFT flavour

L-INS-i

*leave other options in default mode

21. Proceed to concatenation, partitioning, and phylogenetic inference with your preferred tools.

22. References

1. Roncoroni, M.; Gallone, B. Preparing Genomic Data for Phylogeny Reconstruction Available online: https://training.galaxyproject.org/training-material/topics/ecology/tutorials/phylogeny-data-prep/tutorial.html (accessed on 12 March 2025).
2. The Galaxy Community The Galaxy Platform for Accessible, Reproducible and Collaborative Biomedical Analyses: 2022 Update. *Nucleic Acids Research* **2022**, *50*, W345–W351, doi:10.1093/nar/gkac247.
3. Bolger, A.M.; Lohse, M.; Usadel, B. Trimmomatic: A Flexible Trimmer for Illumina Sequence Data. *Bioinformatics* **2014**, *30*, 2114–2120.
4. Andrews, S. FastQC: A Quality Control Tool for High Throughput Sequence Data. *(No Title)* **2010**.
5. Li, D.; Liu, C.-M.; Luo, R.; Sadakane, K.; Lam, T.-W. MEGAHIT: An Ultra-Fast Single-Node Solution for Large and Complex Metagenomics Assembly via Succinct de Bruijn Graph. *Bioinformatics* **2015**, *31*, 1674–1676.
6. Fu, L.; Niu, B.; Zhu, Z.; Wu, S.; Li, W. CD-HIT: Accelerated for Clustering the next-Generation Sequencing Data. *Bioinformatics* **2012**, *28*, 3150–3152.
7. Kyran, A. Fasta Statistics: Display Summary Statistics for a Fasta File 2021.
8. Simão, F.A.; Waterhouse, R.M.; Ioannidis, P.; Kriventseva, E.V.; Zdobnov, E.M. BUSCO: Assessing Genome Assembly and Annotation Completeness with Single-Copy Orthologs. *Bioinformatics* **2015**, *31*, 3210–3212.
9. Gu, Q.; Batut, B.; Soranzo, N.; Gamaleldin, H.; Von Kuster, G. Bgruening/Galaxytools: September Release 2019. *Zenodo: Geneva, Switzerland* **2018**.
10. Smit, A.F.A.; Hubley, R.; Green, P. 2015. RepeatMasker Open-4.0 2013.
11. Keller, O.; Kollmar, M.; Stanke, M.; Waack, S. A Novel Hybrid Gene Prediction Method Employing Protein Multiple Sequence Alignments. *Bioinformatics* **2011**, *27*, 757–763.
12. Klemm, P.; Stadler, P.F.; Lechner, M. Proteinortho6: Pseudo-Reciprocal Best Alignment Heuristic for Graph-Based Detection of (Co-)Orthologs. *Front. Bioinform.* **2023**, *3*, doi:10.3389/fbinf.2023.1322477.
13. Katoh, K.; Standley, D.M. MAFFT Multiple Sequence Alignment Software Version 7: Improvements in Performance and Usability. *Molecular Biology and Evolution* **2013**, *30*, 772–780, doi:10.1093/molbev/mst010.
