# Supplementary figures and images for: Unraveling a 150-Year-Old Enigma: Psalidodon rivularis (Acestrorhamphidae: Acestrorhampinae), a Species Complex or a Polymorphic Species?
Source: Biology (Basel). 2025 Dec 16;14(12):1793. doi: 10.3390/biology14121793 (PMC12730566; doi:10.3390/biology14121793)

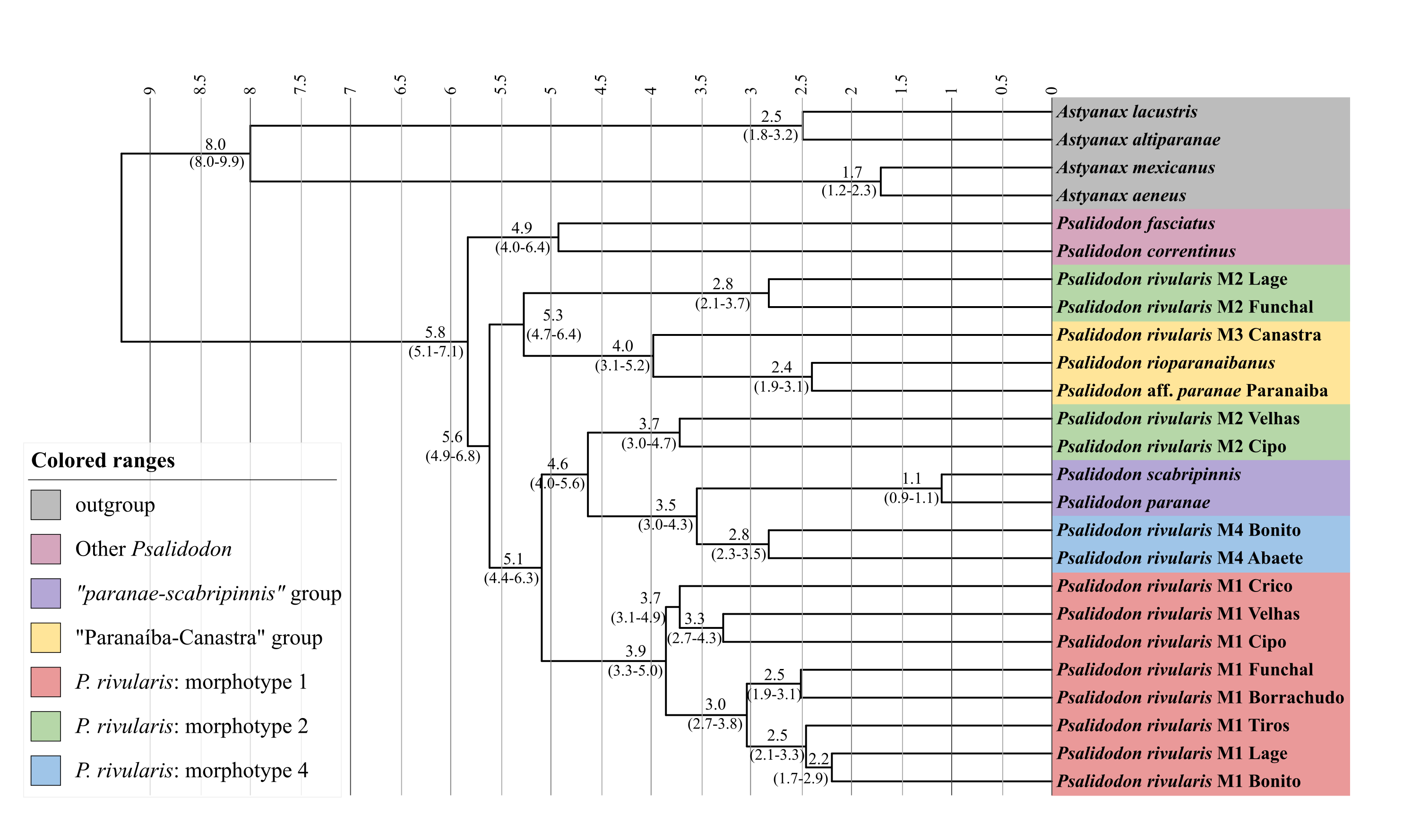

Supplement: Supplementary file 1 [file biology-14-01793-s001.zip › Supplementary Material S6.tiff]
